# Novel Generation of FAP Inhibitor-Based Homodimers for Improved Application in Radiotheranostics

**DOI:** 10.3390/cancers15061889

**Published:** 2023-03-21

**Authors:** Marcel Martin, Sanjana Ballal, Madhav Prasad Yadav, Chandrasekhar Bal, Yentl Van Rymenant, Joni De Loose, Emile Verhulst, Ingrid De Meester, Pieter Van Der Veken, Frank Roesch

**Affiliations:** 1 Department of Chemistry—TRIGA Site, Johannes Gutenberg University Mainz, 55128 Mainz, Germany; froesch@uni-mainz.de; 2 Department of Nuclear Medicine, All India Institute of Medical Sciences, New Delhi 110029, India; mail.sanjanaballal87@gmail.com (S.B.); madhav_yadav2000@yahoo.com (M.P.Y.); csbal@hotmail.com (C.B.); 3 Laboratory of Medical Biochemistry, Department of Pharmaceutical Sciences, University of Antwerp, 2610 Wilrijk, Belgium; yentl.vanrymenant@uantwerpen.be (Y.V.R.); joni.deloose@uantwerpen.be (J.D.L.); emile.verhulst@uantwerpen.be (E.V.); ingrid.demeester@uantwerpen.be (I.D.M.); 4 Laboratory of Medicinal Chemistry, Department of Pharmaceutical Sciences, University of Antwerp, 2610 Wilrijk, Belgium; pieter.vanderveken@uantwerpen.be

**Keywords:** theranostics, radiometals, positron emission tomography (PET), radionuclide therapy (RNT), radioligand therapy (RLT), targeted alpha-particle therapy (TAT), fibroblast activation protein alpha (FAP), FAPi dimers, homodimeric FAPi-based radiopharmaceuticals, medullary thyroid cancer

## Abstract

**Simple Summary:**

Radiopharmaceuticals targeting the fibroblast activation protein alpha (FAP) can be used in many different cancer types since FAP is highly expressed in the tumor microenvironment of almost all epithelial cancers. Monomeric radiotracers have shown great potential in molecular imaging (diagnosis), but the tumor retention time is relatively short (few hours). For effective radioligand therapy (RLT), the biological half-life of the radiotracer should ideally match the physical half-life of the important therapeutic radionuclides ^177^Lu and ^225^Ac (6.7 and 9.9 days). The tumor retention was improved with the FAPi homodimer DOTAGA.(SA.FAPi)_2_. In terms of optimizing, the new FAPi homodimers DO3A.Glu.(FAPi)_2_ and DOTAGA.Glu.(FAPi)_2_.were synthesized. DOTAGA.Glu.(FAPi)_2_ showed superior radiolabeling properties (including successful ^225^Ac-labeling, higher hydrophilicity), in vitro affinity and selectivity compared to DOTAGA.(SA.FAPi)_2_. In addition, significantly reduced uptake in the critical organs (liver, colon) compared to [^177^Lu]Lu-DOTAGA.(SA.FAPi)_2_ was observed with [^177^Lu]Lu-DOTAGA.Glu.(FAPi)_2_ in a first patient study (medullary thyroid cancer) while maintaining high and prolonged tumor uptake.

**Abstract:**

Radiopharmaceuticals based on the highly potent FAP inhibitor (FAPi) UAMC-1110 have shown great potential in molecular imaging, but the short tumor retention time of the monomers do not match the physical half-lives of the important therapeutic radionuclides ^177^Lu and ^225^Ac. This was improved with the dimer DOTAGA.(SA.FAPi)_2_, but pharmacological and radiolabeling properties still need optimization. Therefore, the novel FAPi homodimers DO3A.Glu.(FAPi)_2_ and DOTAGA.Glu.(FAPi)_2_. were synthesized and quantitatively radiolabeled with ^68^Ga, ^90^Y, ^177^Lu and ^225^Ac. The radiolabeled complexes showed high hydrophilicity and were generally stable in human serum (HS) and phosphate-buffered saline (PBS) at 37 °C over two half-lives, except for [^225^Ac]Ac-DOTAGA.Glu.(FAPi)_2_ in PBS. In vitro affinity studies resulted in subnanomolar IC_50_ values for FAP and high selectivity for FAP over the related proteases PREP and DPP4 for both compounds as well as for [^nat^Lu]Lu-DOTAGA.Glu.(FAPi)_2_. In a first proof-of-principle patient study (medullary thyroid cancer), [^177^Lu]Lu-DOTAGA.Glu.(FAPi)_2_ was compared to [^177^Lu]Lu-DOTAGA.(SA.FAPi)_2_. High uptake and long tumor retention was observed in both cases, but [^177^Lu]Lu-DOTAGA.Glu.(FAPi)_2_ significantly reduces uptake in non-target and critical organs (liver, colon). Overall, the novel FAPi homodimer DOTAGA.Glu.(FAPi)_2_ showed improved radiolabeling in vitro and pharmacological properties in vivo compared to DOTAGA.(SA.FAPi)_2_. [^177^Lu]Lu-DOTAGA.Glu.(FAPi)_2_ and [^225^Ac]Ac-DOTAGA.Glu.(FAPi)_2_ appear promising for translational application in patients.

## 1. Introduction

Fibroblast activation protein alpha (FAP), also called Seprase, is a S9B family serine protease and an integral type-II transmembrane glycoprotein [1]. It is mainly expressed on activated fibroblasts such as cancer-associated fibroblasts (CAFs), fibroblasts present during wound healing [2], at sites of inflammation (e.g., arthritis) [3] and fibrosis (e.g., liver cirrhosis) [4]. However, FAP is absent in resting fibroblasts of normal healthy tissue. CAFs behave as activated stromal fibroblasts and occupy a major part of the tumor microenvironment (TME) that further consists of vascular, inflammatory and immune cells as well as extracellular matrix components (ECM) [5]. The TME plays an essential role in tumorigenesis, tumor growth, metastasis and angiogenesis and can account for up to 90% of the total tumor mass [5,6,7,8]. Each epithelial tumor more than 1 mm in diameter consists of an extensive TME. More than 90% of these epithelial tumors strongly express FAP, including prostate, breast, lung, ovarian and colorectal cancer, among others [6].

FAP is only enzymatically active as a dimer and has unique dual enzymatic activity, both dipeptidyl peptidase and prolyl endopeptidase, whereas the similar serine proteases prolyl endopeptidase (PREP) and other post-proline dipeptidyl peptidases (DPP4, DPP8 and DPP9) only have one of these activities [8,9,10,11,12]. Natural substrates have been identified including several neuropeptides (neuropeptide Y, peptide YY etc.) [13], α_2_-antiplasmin [4,14] and denatured type I collagen [15,16]. The actual in vivo role of FAP is not fully understood yet but seems to be non-essential in healthy tissue [8]. In contrast, FAP is considered to play an important role in fibroblast growth, tissue remodeling of the ECM, epithelial carcinogenesis, tumor growth (proliferation) and angiogenesis in malignant tissue [8,17,18,19,20,21,22].

Compared to other tumor targets that are vastly used in nuclear medicine such as prostate-specific membrane antigen (PSMA) or somatostatin receptor (SSTR), radiopharmaceuticals targeting FAP do not address the cancer cells directly but indirectly via its TME. PSMA inhibitor-based tracers can basically only be applied in prostate cancer and octreotide-based SSTR-targeting tracers for neuroendocrine tumors (NETs), while targeting FAP allows for a broader application in many different types of cancers, which makes it a more universal pan-tumor target. Jeremie Calais discussed FAP as potentially being “the next billion dollar nuclear theranostics target” following [^177^Lu]Lu-PSMA-617 and [^177^Lu]Lu-DOTA-TATE that has had a great impact in nuclear medicine in the last years [23].

**Figure 1:**
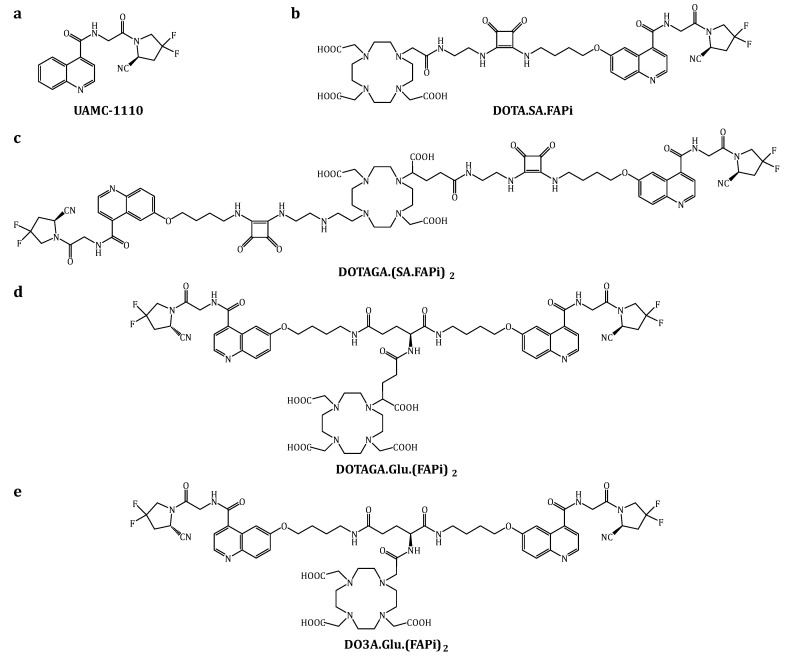
Structures of the original FAP inhibitor UAMC-1110 (**a**), the FAPi-based monomer DOTA.SA.FAPi (**b**), the first-generation linear FAPi dimer DOTAGA.(SA.FAPi)_2_ (**c**) and the second-generation FAPi dimers DOTAGA.Glu.(FAPi)_2_ (**d**) and DO3A.Glu.(FAPi)_2_ (**e**).

The FAP inhibitor (FAPi) UAMC-1110 (Figure 1a) was the first small molecule that showed high affinity for FAP and high selectivity over PREP and the DPPs [24]. Since then, several FAPi-based radiopharmaceuticals have been synthesized [25,26,27,28,29,30,31,32,33,34]. Some of them have already been systematically investigated in human positron emission tomography (PET) studies such as FAPI-04, FAPI-46, and DOTA.SA.FAPi (Figure 1b) [35,36,37,38,39,40].

Most of them are conjugated with bifunctional chelators such as DOTA to enable PET imaging with ^68^Ga. The rapid renal clearance of monomers such as the squaramide (SA)-based [^68^Ga]Ga-DOTA.SA.FAPi or [^68^Ga]Ga-FAPI-04 resulted in high-contrast PET images in various cancers even at early time points (10 min p.i.) [25,40]. However, the short tumor retention time and as such the biological half-life does not match the physical half-life of therapeutic radionuclides such as ^177^Lu (*t*_1/2_ = 6.7 d) or ^225^Ac (*t*_1/2_ = 9.9 d), which limits the use of these compounds for radioligand therapy (RLT) with the beta minus particle emitter ^177^Lu or targeted alpha-particle therapy (TAT) with the alpha emitter ^225^Ac. 

Recently, peptides were introduced as FAP-targeting vectors in compounds such as FAP-2286, which showed prolonged retention in CAFs-expressing tissue and which represent a new approach regarding FAP radiotherapeutics with a similar approach to TOC or TATE used for peptide receptor radionuclide therapy by targeting SSTRs of NETs [41,42].

Another approach is to apply the dimeric concept, which has already shown, to improve accumulation and to prolong tumor retention time in the context of different targets (e.g., PSMA) beforehand [43,44,45,46,47]. This has led to the development of the first homodimeric structure DOTAGA.(SA.FAPi)_2_ (see Figure 1c). Other groups have adapted this approach, and several other dimeric FAPi-based radiopharmaceuticals were published recently [48,49,50,51]. Zhao et al. developed the dimer DOTA-2P(FAPI)_2_ based on the FAPI-46 structure, and the ^68^Ga-labeled derivative was investigated in vivo [48,49]. Galbiati et al. developed the dimeric BiOncoFAP-DOTAGA [50]. The publication mainly focused on the ^177^Lu-labeled therapeutic agent that showed promising results in preclinical studies. Both studies confirmed that the homodimeric concept is a successful strategy to target FAP and to prolong tumor retention as first demonstrated with DOTAGA.(SA.FAPi)_2_.

The ^177^Lu-labeled derivative [^177^Lu]Lu-DOTAGA.(SA.FAPi)_2_ has already been investigated extensively in RNT of radioiodine-refractory differentiated thyroid cancer (RR-DTC) patients, among others (e.g., breast cancer) [39,52]. Much longer tumor residence times of up to one week could be measured via planar scintigraphy and single-photon emission computed tomography (SPECT) imaging [39]. The colon was identified as the critical organ regarding the radiation dose due to slow biliary excretion [39,52]. Furthermore, the linear molecular design with the central chelator may have resulted in steric hindrance during complexation, which led to slower reaction kinetics and/or lower radiochemical yields, in particular with ^225^Ac.

On the one hand, the new compounds DO3A.Glu.(FAPi)_2_ and DOTAGA.Glu.(FAPi)_2_ (Figure 1d,e) were designed, synthesized and investigated to improve radiolabeling, especially regarding ^225^Ac and therefore allowing for potential application in TAT. On the other hand, the pharmacokinetic and pharmacodynamic properties should be optimized while maintaining or even further improving the desired long tumor retention time. In contrast to the original linear dimer DOTAGA.(SA.FAPi)_2_, these new FAPi dimers are designed in a branched structure where the two FAPi targeting vectors and the terminal chelator are coupled via a central glutamic acid (Glu) linker. The further use of squaramide as a linker or spacer was renounced with the intention to synthesize more hydrophilic compounds instead. This could accelerate clearance from non-target tissues as well as excretion to reduce the radiation dose for healthy organs such as colon and liver.

Following the organic synthesis of the precursors, the radiolabeling properties with different radiometals (^68^Ga, ^177^Lu, ^225^Ac and ^90^Y) as well as the complex stabilities were investigated. In addition, the lipophilicity of the ^68^Ga- and ^177^Lu-labeled derivatives was evaluated. The new FAPi dimers were tested for their in vitro affinity for FAP as well as PREP, DPP4, DPP8, and DPP9. Beyond that, first proof-of-concept patient investigations were carried out with [^177^Lu]Lu-DOTAGA.Glu.(FAPi)_2_, which demonstrated faster excretion kinetics and therefore reduced the radiation dose for healthy organs such as the colon and liver. All these studies were performed in comparison to DOTAGA.(SA.FAPi)_2_. 

## 2. Materials and Methods

### 2.1. Chemicals and Equipment

All basic chemicals were purchased from Sigma-Aldrich and Merck (Darmstadt, Germany), TCI (Eschborn, Germany), ABCR (Karlsruhe, Germany), Alfa Aesar by Thermo Fisher Scientific (Kandel, Germany), Acros Organics (Schwerte, Germany) and VWR (Bruchsal, Germany). Dry solvents were bought from Acros Organics, Alfa Aesar by Thermo Fischer Scientific and Merck and deuterated solvents from Deutero (Kastellaun, Germany). The chelators DOTA-tri(^t^Bu)ester **8** (2-(4,7,10-tris(2-(*tert*-butoxy)-2-oxoethyl)-1,4,7,10-tetraazacyclododecan-1-yl)acetic acid) and DOTA-GA(^t^Bu)_4_ **5** (5-(*tert*-butoxy)-5-oxo-4-(4,7,10-tris(2-(*tert*-butoxy)-2-oxoethyl)-1,4,7,10-tetra-azacyclododecan-1-yl)pentanoic acid) were obtained from CheMatech (Dijon, France). (*S*)-6-(4-aminobutoxy)-*N*-(2-(2-cyano-4,4-difluoropyrrolidin-1-yl)-2-oxoethyl)quinoline-4-car-boxamide (FAPi-NH_2_ **1**) was purchased from KE Biochem Co. (Shangai, China) or synthesized according to Figure S1. 

Thin-layer chromatography (TLC) plates coated with silica gel 60 F_254_ from Merck were used. For analysis, they were investigated via UV lamp (λ = 254 and 366 nm) as well as staining with potassium permanganate. Silica 60 (0.040–0.063 mm particle size) was used for column chromatography. 

Nuclear magnetic resonance (NMR) spectra were recorded in deuterated solvents on an Avance II 400 spectrometer (400 MHz, 5 mm BBFO sample head with z-gradient and ATM, SampleXPress 60 sample changer) or an Avance III 300 spectrometer (300 MHz, 5 mm BBFO sample head with z-gradient and ATM, BACS 60 sample changer) from Bruker (Rheinstätten, Germany). MestReNova 14.2.0 software from Mestrelab Research (Santiago de Compostela, Spain) was used to analyze the spectra. 

Liquid chromatography coupled with electrospray ionization mass spectrometry (ESI-LC/MS) was measured using Agilent Technologies 1220 Infinity LC coupled to an Agilent Technologies 6130B Single Quadruple LC/MS system with an Agilent Zorbax SB-C18 column (21 × 50 mm, 1.8 µm) with linear gradients of acetonitrile (MeCN)/Milli-Q^®^ water (H_2_O) + 0.05% formic acid (HFo) and a flow rate of 0.5 mL/min. 

High-pressure liquid chromatography coupled with electrospray ionization mass spectrometry (ESI-HPLC-MS) measurements were performed using an Agilent Technologies G6545A Q-ToF with electron spray ionization coupled to a 1260 Infinity II HPLC system (Agilent Technologies) with G7111B 1260 Quaternary Pump, G7129A 1260 Vial Sampler, and G7116A Multicolumn Thermostat. Separation was performed using an Agilent Poroshell 120 EC-C8 column (2.1 × 100 mm, 2.7 µm) with H_2_O + 2% MeCN/MeCN + 2% H_2_O + 0.05% Hfo and a flow rate of 0.1 mL/min. 

Semi-preparative reversed-phase high-pressure liquid chromatography (RP-HPLC) was performed using LaChrom HPLC (7000 series) from Merck Hitachi with L-7100 pump, L-7400 UV detector (λ = 254 nm), a D-7000 interface and autosampler. Separation was performed using a Phenomenex Synergi Max-RP C18 column (250 × 10 mm, 4 µm) and linear gradient of MeCN/H_2_O + 0.1% trifluoacetic acid (TFA) and a flow rate of 5 mL/min.

For radiolabeling, only chemicals of the highest purity (TraceSelect if available) were used. Radio-TLCs were developed using 0.1 M citrate buffer (pH = 4.0). Additional TLCs were developed using 1 M ammonium acetate (pH = 4.0)/methanol (AmOAc/MeOH, 1:1). All radio-TLCs were evaluated using a CR-35 Bio Test-Imager from Duerr-ndt (Bietigheim-Bissingen, Germany) and AIDA software from Elysia-Raytest (Straubenhardt, Germany). Some radio-TLCs of ^225^Ac-labelings were additionally analyzed via high-resolution gamma spectroscopy with a HPGe detector. Analytical radio-HPLC was performed also using a Merck Hitachi LaChrom HPLC (7000 series). Separation was performed using Phenomenex Luna C18 column (250 × 4.6 mm, 5 µm) and linear gradient of MeCN/H_2_O + 0.1% TFA and flow rate of 1 mL/min. The radio-HPLC is equipped with a Ramona analog radio detector from Elysia Raytest with its energy window set to 100–1200 keV for ^68^Ga and 100–250 keV for ^177^Lu.

### 2.2. Organic Synthesis

**Figure 2:**
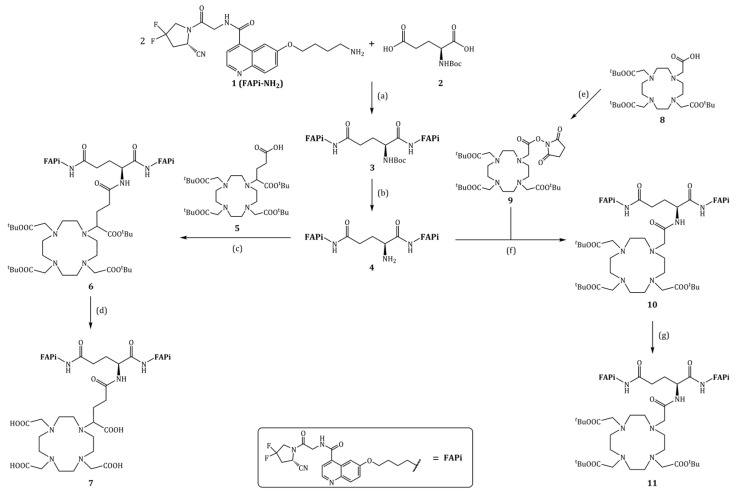
Organic synthesis of DOTAGA.Glu.(FAPi)_2_ **7** and DO3A.Glu.(FAPi)_2_ **11**: (**a**) HOBt, EDC*HCl, DIPEA, DMF, RT, 1 d, 74%; (**b**) 4 M HCl in 1,4-dioxane, MeCN, 0 °C–RT, 6 h, 95%; (**c**) HATU, DIPEA, DMF, 30 °C, 3 d; (**d**) TFA:MeCN:TIPS:H_2_O (85:10:5:2.5), RT, 5 h, 28% (over 2 steps); (**e**) NHS, HBTU, MeCN, DMF, 30 °C, 1 d, 96%; (**f**) DIPEA, DMF, 40 °C, 1 d; (**g**) TFA:TIPS:H_2_O (95:2.5:2.5), RT, 8 h, 29% (over 2 steps).

Figure 2 shows the synthesis scheme of the precursors DOTAGA.Glu.(FAPi)_2_ **7** and DO3A.Glu.(FAPi)_2_ **11**.

#### 2.2.1. Synthesis of Glu.(FAPi)_2_

##### tert-Butyl ((S)-1,5-bis((4-((4-((2-((S)-2-cyano-4,4-difluoropyrrolidin-1-yl)-2-oxoethyl)carbamoyl)-quinolin-6-yl)oxy)butyl)amino)-1,5-dioxopentan-2-yl)carbamate (**3**, Boc-Glu.(FAPi)_2_)

*N*-*tert*-Butoxycarbonyl-L-glutamic acid **2** (Boc-Glu-OH, 49.5 mg, 200 µmol, 1.00 eq), 1-hydroxybenzotriazole (HOBt, 70 mg, 518 µmol, 2.59 eq) and 1-ethyl-3-(3-dimethylaminopropyl)carbodiimide hydrochloride (EDC*HCl, 100 mg, 522 µmol, 2.61 eq) were dissolved in dry *N,N*-dimethylformamide (DMF, 4 mL), and *N,N*-diisopropylethylamine (DIPEA, 17.0 µL, 100 µmol, 0.50 eq) was added under argon atmosphere. The solution was stirred at room temperature (RT) for one hour. Then, a solution of FAPi-NH_2_ **1** (173 mg, 400 µmol, 2.00 eq) and DIPEA (102 µL, 600 µmol, 3.00 eq) in DMF (2 mL) was added. After another 4 h at RT, further HOBt (13.5 mg, 100 µmol, 0.50 eq) and EDC*HCl (19.2 mg, 100 µmol, 0.50 eq) were added, and after another 30 min, a solution of FAPi-NH_2_ **1** (43.4 mg, 100 µmol, 0.50 eq) and DIPEA (17.0 µL, 100 µmol, 0.50 eq) in DMF (1 mL) was added again. This addition of HOBt, EDC*HCl (0.50 eq each) followed by FAPi-NH_2_ (28.5 mg, 66 µmol, 0.33 eq) and DIPEA (0.50 eq) was repeated one more time, and after 4 more hours, the solvent was removed in vacuo. After column chromatography (CHCl_3_/MeOH, 100:10–15)) **3** was obtained as a yellowish solid (158 mg, 147 µmol, 74%). ^1^H-NMR (400 MHz, DMF-d_7_): δ [ppm] = 8.98 (t, *J* = 6.0 Hz, 1H), 8.83 (d, *J* = 4.3 Hz, 1H), 8.29–8.17 (m, 1H), 8.03–7.94 (m, 3H), 7.93–7.87 (m, 1H), 7.80–7.71 (m, 1H), 7.58 (d, *J* = 4.3 Hz, 1H), 7.52–7.43 (m, 2H), 7.34 (dd, *J* = 8.5, 2.0 Hz, 1H), 7.25 (dd, *J* = 8.4, 2.5 Hz, 1H), 6.83 (d, *J* = 7.8 Hz, 1H), 5.31 (dd, *J* = 9.6, 2.5 Hz, 1H), 4.53–3.70 (m, 11H), 3.37–3.01 (m, 8H), 2.33–2.25 (m, 2H), 2.16–1.83 (m, 4H), 1.78–1.66 (m, 2H), 1.62–1.44 (m, 2H), 1.42 (s, 9H), 1.40–1.37 (m, 6H). ^19^F-NMR (300 MHz, DMF-d_7_): δ [ppm] = −74.48 (s). MS (ESI^+^): *m*/*z* (%) = 487.8 (100, [M−Boc+H]^2+^), 537.8 (73, [M+H]^2+^), 1074.4 (9, [M+H]^+^), 1075.4 (6, [M+H]^+^), calculated for C_52_H_59_F_4_N_11_O_10_: 1073.44 [M].

##### (S)-2-Amino-N^1^,N^5^-bis(4-((4-((2-((S)-2-cyano-4,4-difluoropyrrolidin-1-yl)-2-oxoethyl)carbamoyl)-quinolin-6-yl)oxy)butyl)pentanediamide (**4**, Glu.(FAPi)_2_)

Boc-Glu.(FAPi)_2_ **3** (158 mg, 147 µmol, 1.00 eq) was dissolved in dry MeCN (3 mL) and under argon atmosphere and at 0 °C, 4 M hydrochloric acid (HCl) in 1,4-dioxane (300 µL, 1.20 mmol, 8.57 eq) was added. The reaction was allowed to warm to RT, and after 6 h, it was diluted with MeOH (20 mL), and the solvent was completely removed in vacuo. Glu.(FAPi)_2_ **4** (136 mg, 140 µmol, 95%) was obtained as a yellowish solid. ^1^H-NMR (300 MHz, MeOD): δ [ppm] = 8.84 z(dd, *J* = 8.4, 4.9 Hz, 2H), 8.01 (dd, *J* = 8.8, 2.6 Hz, 4H), 7.72 (dd, *J* = 6.6, 4.8 Hz, 2H), 7.55 (ddd, *J* = 9.3, 4.6, 2.7 Hz, 2H), 5.16 (dt, *J* = 9.3, 3.4 Hz, 2H), 4.35 (d, *J* = 2.1 Hz, 4H), 4.31–4.07 (m, 8H), 3.91 (t, *J* = 6.2 Hz, 1H), 3.58–3.36 (m, 2H), 3.11–2.71 (m, 6H), 2.47–2.37 (m, 2H), 2.17–2.00 (m, 2H), 2.00–1.87 (m, 4H), 1.86–1.70 (m, 4H). ^19^F-NMR (300 MHz, MeOD): δ [ppm] = −77.29 (s). MS (ESI^+^): *m*/*z* (%) = 325.6 (100, [M−Boc+H]^3+^), 487.8 (28, [M+H]^2+^), 974.3 (5, [M+H]^+^), calculated for C_47_H_51_F_4_N_11_O_8_: 973.39 [M].

#### 2.2.2. Synthesis of DOTAGA.Glu.(FAPi)_2_

tri-tert-Butyl 2,2′,2″-(10-(5-(((S)-1,5-bis((4-((4-((2-((S)-2-cyano-4,4-difluoropyrrolidin-1-yl)-2-oxo-ethyl)carbamoyl)quinolin-6-yl)oxy)butyl)amino)-1,5-dioxopentan-2-yl)amino)-1-(tert-butoxy)-1,5-dioxopentan-2-yl)-1,4,7,10-tetraazacyclododecane-1,4,7-triyl)triacetate (**6**, DOTAGA(^t^Bu)_4_.Glu.(FAPi)_2_)

DOTAGA(^t^Bu)_4_ **5** (56.1 mg, 80.0 µmol, 1.15 eq), 1-[bis(dimethylamino)methylene]-1*H*-1,2,3-triazolo [4,5-b]pyridinium 3-oxide hexafluorophosphate (HATU, 30.3 mg, 80.0 µmol, 1.15 eq) and DIPEA (15.0 µL, 88 µmol, 1.25 eq) were dissolved in dry DMF (1 mL) under argon atmosphere and stirred at 30 °C for one hour. A solution of Glu.(FAPi)_2_ (68.0 mg, 70.0 µmol, 1.00 eq) and DIPEA (30.0 µL, 175 µmol, 2.50 eq) in dry DMF (1 mL) was added. It was stirred for two days at 30 °C, and then, more HATU (15.2 mg, 40.0 µmol, 0.50 eq) was added. After another day of stirring at 30 °C, the solvent was removed in vacuo. A yellow oil was obtained, which was directly used in the next step without further purification. MS (ESI^+^): *m*/*z* (%) = 414.97 (13, [M+H]^4+^), 415.22 (12, [M+H]^4+^), 552.95 (100, [M+H]^3+^), 553.29 (97, [M+H]^3+^), 553.62 (51, [M+H]^3+^), 553.96 (18, [M+H]^3+^), 828.93 (82, [M+H]^2+^), 829.43 (78, [M+H]^2+^), 829.93 (40, [M+H]^2+^), 830.43 (15, [M+H]^2+^), 1656.85 (87, [M+H]^+^), 1657.85 (85, [M+H]^+^), 1658.85 (43, [M+H]^+^), 1659.86 (15, [M+H]^+^), calculated for C_82_H_113_F_4_N_15_O_17_: 1655.84 [M].

2,2′,2″-(10-(4-(((S)-1,5-bis((4-((4-((2-((S)-2-Cyano-4,4-difluoropyrrolidin-1-yl)-2-oxoethyl)-carbamoyl)quinolin-6-yl)oxy)butyl)amino)-1,5-dioxopentan-2-yl)amino)-1-carboxy-4-oxobutyl)-1,4,7,10-tetraazacyclododecane-1,4,7-triyl)triacetic acid (**7**, DOTAGA.Glu.(FAPi)_2_)

DOTAGA(^t^Bu)_4_.Glu.(FAPi)_2_ **6** was dissolved in TFA (850 µL), MeCN (100 µL), triisopropylsilane (TIPS, 50 µL) and H_2_O (25 µL) and stirred at RT for 5 h. After co-distillation with MeOH (5 × 10 mL) and evaporation to dryness, the crude product was purified by semipreparative RP-HPLC (22–24% MeCN in 20 min, *t_R_* = 17–18 min), and **7** was obtained as a yellow solid (28.0 mg, 19.5 µmol, 28%). MS (ESI^+^): *m*/*z* (%) = 358.85 (65, [M+H]^4+^), 369.05 (24, [M+MeCN+H]^4+^), 478.30 (100, [M+H]^3+^), 717.30 (6, [M+H]^2+^), 1432.40 (1, [M+H]^+^), 1454.70 (1, [M+Na]^+^), calculated for C_66_H_81_F_4_N_15_O_17_: 1431.59 [M].

#### 2.2.3. Synthesis of DO3A.Glu.(FAPi)_2_

tri-tert-Butyl 2,2′,2″-(10-(2-((2,5-dioxopyrrolidin-1-yl)oxy)-2-oxoethyl)-1,4,7,10-tetraazacyclodo-decane-1,4,7-triyl)triacetate (**9**, DOTA(^t^Bu)_3_-NHS)

DOTA-tris(*tert*-butyl ester) **8** (573 mg, 1.00 mmol, 1.00 eq) and HBTU (417 mg, 1.10 mmol, 1.10 eq) were dissolved in dry MeCN (12 mL) and dry DMF (5 mL), and then, *N*-hydroxysuccinimide (NHS, 127 mg, 1.10 mmol, 1.10 eq) was added. After stirring at 30 °C for 6 h, 2-(1*H*-benzotriazol-1-yl-)-1,1,3,3-tetramethyluronium hexafluorophosphate (HBTU, 190 mg, 501 µmol, 0.50 eq) and NHS (57.5 mg, 500 µmol, 0.50 eq) were added, and stirring was continued overnight. After all solvents were removed in vacuo, purification by column chromatography (dichloromethane/MeOH (100:10)) gave **9** as a colorless solid (643 mg, 961 μmol, 96%). ^1^H-NMR (400 MHz, CDCl3): δ [ppm] = 2.97–2.77 (m, 12H), 2.66–2.02 (m, 16H), 1.44 (s, 27H). MS (ESI^+^): *m*/*z* (%) = 335.7 (100, [M+H]^2+^), 670.4 (50, [M+H]^+^), 671.4 (18, [M+H]^+^), calculated for C_32_H_55_N_5_O_10_: 669.39 [M].

tri-tert-Butyl 2,2′,2″-(10-(2-(((S)-1,5-bis((4-((4-((2-((S)-2-cyano-4,4-difluoropyrrolidin-1-yl)-2-oxo-ethyl)carbamoyl)quinolin-6-yl)oxy)butyl)amino)-1,5-dioxopentan-2-yl)amino)-2-oxoethyl)-1,4,7,10-tetraazacyclododecane-1,4,7-triyl)triacetate (**10**, DOTA(tBu)_3_.Glu.(FAPi)_2_)

DOTA(^t^Bu)_3_-NHS **9** (40.2 mg, 60.0 µmol, 1.20 eq) and Glu.(FAPi)_2_ **4** (48.7 mg, 50.0 µmol, 1.00 eq) were dissolved in dry DMF (2 mL), and DIPEA (200 µL) was added. After stirring at 40 °C under argon atmosphere for one day, the solvents were completely removed in vacuo. A yellow oil was obtained and directly used in the next step without further purification. MS (ESI^+^): *m*/*z* (%) = 382.95 (22, [M+H]^4+^), 383.20 (19, [M+H]^4+^), 491.57 (34, [M−^t^Bu+H]^3+^), 491.90 (28, [M−^t^Bu+H]^3+^), 492.24 (13, [M−^t^Bu+H]^3+^), 510.26 (100, [M+H]^3+^), 510.59 (90, [M+H]^3+^), 510.93 (44, [M+H]^3+^), 511.26 (14, [M+H]^3+^), 764.88 (42, [M+H]^2+^), 765.38 (37, [M+H]^2+^), 765.89 (17, [M+H]^2+^), 1528.76 (25, [M+H]^+^), 1529.76 (22, [M+H]^+^), 1530.77 (10, [M+H]^+^), calculated for C_75_H_101_F_4_N_15_O_15_: 1527.75 [M].

2,2′,2″-(10-(2-(((S)-1,5-bis((4-((4-((2-((S)-2-Cyano-4,4-difluoropyrrolidin-1-yl)-2-oxoethyl)-carbamoyl)quinolin-6-yl)oxy)butyl)amino)-1,5-dioxopentan-2-yl)amino)-2-oxoethyl)-1,4,7,10-tetraazacyclododecane-1,4,7-triyl)triacetic acid (**11**, DO3A.Glu.(FAPi)_2_)

DOTA(^t^Bu)_3_.Glu.(FAPi)_2_ **10** was dissolved in TFA (1.9 mL), TIPS (50 µL) and H_2_O (50 µL) and stirred at RT for 8 h. After co-distillation with MeOH (4 × 10 mL), the solvents were completely removed under reduced pressure again. After semipreparative RP-HPLC (22–23% MeCN in 16 min, *t_R_* = 14–15 min), **11** was obtained as a yellow solid (19.9 mg, 14.6 µmol, 29%). MS (ESI^+^): *m*/*z* (%) = 340.85 (42, [M+H]^4+^), 351.00 (57, [M+MeCN+H]^4+^), 361.35 (13, [M+2MeCN+H]^4+^), 454.15 (100, [M+H]^3+^), 468.00 (20, [M+MeCN+H]^3+^), 680.85 (9, [M+H]^2+^), calculated for C_63_H_77_F_4_N_15_O_15_: 1359.57 [M].

#### 2.2.4. Synthesis of ^nat^Lu-Complexes

##### [^nat^Lu]Lu-DOTAGA.Glu.(FAPi)_2_ (**^nat^Lu-7**)

DOTAGA.Glu.(FAPi)_2_ **7** (8.6 mg, 6.0 µmol, 1.00 eq) was dissolved in 500 µL 1 M HEPES (pH = 5.5), 100 µL 1 M ammonium acetate (pH = 5.5) and 200 µL EtOH. Then, 0.1 M LuCl_3_ (150 µL, 15.0 µmol, 2.50 eq) solution in 1 M ammonium acetate (pH = 5.5) was added and shaken at 90 °C for 6 h. Subsequent semi-preparative RP-HPLC (22–24% MeCN in 12 min, *t_R_* = 11.5 min) yielded [^nat^Lu]Lu.DOTAGA.Glu.(FAPi)_2_ as a yellow solid (5.3 mg, 3.3 µmol, 55%). MS (ESI^+^): *m*/*z* (%) = *m*/*z* (%) = 535.50 (100, [M+H]^3+^), 802.95 (36, [M+H]^2+^), calculated for C_66_H_78_F_4_LuN_15_O_17_: 1603.50 [M].

##### [^nat^Lu]Lu-DO3A.Glu.(FAPi)_2_ (**^nat^Lu-11**)

DO3A.Glu.(FAPi)_2_ **11** (2.8 mg, 2.0 µmol, 1.00 eq) was dissolved in 500 µL of 1 M HEPES buffer (pH = 5.5). Then, 0.1 M LuCl_3_ solution (40 µL, 4.0 µmol, 2.00 eq) was added and shaken at 90 °C for 4 h. Subsequent semi-preparative RP-HPLC (20–25% MeCN in 20 min, *t_R_* = 14–15 min) yielded [^nat^Lu]Lu-DO3A.Glu.(FAPi)_2_ as a yellow solid (0.7 mg, 0.46 µmol, 23%). MS (ESI^+^): *m*/*z* (%) = 511.55 (100, [M+H]^3+^), 766.75 (14, [M+H]^2+^), calculated for C_63_H_74_F_4_LuN_15_O_15_: 1531.48 [M].

### 2.3. Radiosynthesis

#### 2.3.1. ^68^Ga-Radiolabeling

Gallium-68 was eluted from a ITM (Isotope Technologies Munich AG, Germany) ^68^Ge/^68^Ga-generator with 5 mL of 0.05 M HCl (TraceSelect). For the labeling of DOTAGA.Glu.(FAPi)_2_ **7**, the eluate (100–400 MBq in 0.5–2.0 mL) was directly used. The equivalent volume of 1 M 4-(2-hydroxyethyl)-1-piperazineethansulfonic acid (HEPES, pH = 4.5) was added, followed by precursor **7** (10–40 nmol, 10–40 µL 1 mM stock solution).

For DO3A.Glu.(FAPi)_2_ **11**, cationic post processing of the ^68^Ge/^68^Ga-generator was carried out according to the procedure developed by Eppard et al. [53]. Precursor **11** (5–20 nmol, 5–20 µL 1 mM stock solution) was added to 400 µL 1 M HEPES (pH = 4.5 or 5.5) buffer solution followed by the addition of gallium-68 (ca. 100 MBq).

The reaction solutions were heated at 95 °C, and reaction controls were taken at different time points to investigate reaction kinetics analyzed via radio-TLC. Additional AmOAc/MeOH (1:1) radio-TLCs and radio-HPLC were carried out for the last reaction control to double-check radiochemical conversion (RCC) and purity (RCP).

#### 2.3.2. ^177^Lu-Radiolabeling

[^177^Lu]LuCl_3_ in 0.04 M HCl) was purchased from ITM (Isotope Technologies Munich SE, Munich, Germany). Then, 400 µL 1 M HEPES (pH = 5.5) buffer solution and the respective quantity of precursor (1 mM stock solution) were added to lutetium-177 (50–100 MBq) and heated at 95 °C. Reaction controls were taken at different time points to investigate reaction kinetics analyzed via radio-TLC. Additional AmOAc/MeOH (1:1) radio-TLCs and radio-HPLC were carried out for the last reaction control to double-check radiochemical conversion (RCC) and purity (RCP).

#### 2.3.3. ^90^Y-Radiolabeling

[^90^Y]YCl_3_ (in 0.04 M HCl) was purchased from EZAG (Eckert & Ziegler Strahlen- und Medizintechnik AG, Berlin, Germany). Then, 400 µL 1 M HEPES (pH = 4.5) buffer solution and DO3A.Glu.(FAPi)_2_ (5 or 10 nmol, 5 or 10 µL of 1 mM stock solution) were added to yttrium-90 (30–60 MBq) and heated at 95 °C. Reaction controls were taken at different time points to investigate reaction kinetics analyzed via radio-TLC.

#### 2.3.4. ^225^Ac-Radiolabeling

[^225^Ac]AcCl_3_ (in 0.04 M HCl) was purchased from ITM (Isotope Technologies Munich SE, Munich, Germany).

Small scale labeling was carried out as follows: ca. 500 kBq [^225^Ac]AcCl_3_ (in 50 µL 0.04 M HCl) was added to 500 µL 0.1 M sodium ascorbate (pH = 7.0) buffer solution, and then, the respective quantity of precursor **11** (10 or 20 nmol, 10 or 20 µL of 1 mM stock solution) was added and heated at 95 °C for 60 min.

Bigger scale labeling was carried out similarly: 1.6–3.2 MBq [^225^Ac]AcCl_3_ (in 100 µL 0.04 M HCl) was added to 1000 µL 0.1 M sodium ascorbate (pH = 7.0) buffer solution, and then, the respective quantity of precursor **11** (30–40 nmol/MBq from 1 mM stock solution) was added and heated at 95 °C for 60 min.

Reaction controls were taken at different time points to investigate reaction kinetics. The radio-TLCs were dried with a heating gun and imaged at different time points (1 h, 1 d) after the TLC had been developed. Additional AmOAc/MeOH (1:1) radio-TLCs were developed for the last reaction control.

Subsequent purification using SepPak^®^ Light C18 cartridge yielded the product. The cartridge was conditioned with 2 mL EtOH and 5 mL H_2_O. The reaction solution was pushed through the cartridge. The reaction vial was rinsed with 0.1 M sodium ascorbate, and the step was repeated once. The product was washed with 1 mL H_2_O and eluted with 2 mL EtOH/saline (1:1). It was diluted with 8 mL of saline containing 100 mg sodium ascorbate to give the final formulation. The radiochemical yield (>98%) was determined via radio-TLC and high-resolution gamma spectroscopy with HPGe detector. For the gamma spectroscopy, a TLC was developed in standard 0.1 M citrate buffer, cut into two pieces at R_f_ = 0.2–0.3 and measured separately after one hour for 15 min each. The integral of the ^221^Fr line (*E_γ_* = 218 keV) of each of the two pieces was considered for the RCC determination.

#### 2.3.5. Complex Stability Measurements

After radiolabeling was carried out (RCP > 95%), the stability in human serum (HS) and phosphate-buffered saline (PBS) was investigated (n = 3) by incubating ca. 10 MBq of the labeled tracer solution in 0.5 mL HS and PBS at 37 °C for 120 min (^68^Ga), for 6 d (^90^Y) and 14 d (^177^Lu). The complex stability was determined via radio-TLC with 0.1 M citrate buffer (pH = 4.0) as the mobile phase.

For ^225^Ac, 350–400 kBq was incubated in 0.5 mL HS and PBS as well as 200 kBq in 1 mL final formulation solution (“pure”). The complex stability was investigated over 20 days, and the citrate radio TLCs were imaged 1 d after they were developed.

#### 2.3.6. Determination of logD_7.4_ (Lipophilicity Measurement)

After radiolabeling was carried out (RCP > 95%), the log*D*_7.4_ value was determined by diluting 10 MBq of the labeled tracer solution to 700 µL with PBS (n = 4). To each, 700 µL of 1-octanol was added and shaken vigorously for 1–2 min (1500 rpm) followed by centrifugation for 1–2 min. Then, 400 µL of the organic and aqueous phase were separated into new Eppendorf tubes. Samples of 3 µL (PBS) or 6 µL (1-octanol) were pipetted onto a TLC plate. Since most of the activity was in the aqueous phase, this was diluted to 700 µL with PBS, and again, 700 µL of 1-octanol was added. The procedure was repeated twice. The TLC plate was imaged (exposure time: 5–10 min), and the integral of each spot (octanol phase: *I_O_*, aqueous PBS phase: *I_PBS_*) was determined. The log*D*_7.4_ value was calculated using Equation (1) where the different volumes of V*_O_* = 6 µL und V*_PBS_* = 3 µL were taken into account.

(1) $$\log D_{7.4}=\log\left(\frac{I_{O}}{2\cdot I_{PBS}}\right)$$

Only the values of the second and third extraction were considered in the calculation.

### 2.4. In Vitro Inhibition Assays (IC_50_ Measurements)

rhFAP (recombinant human fibroblast activation protein), DPP8 (dipeptidyl peptidase VIII) and DPP9 (dipeptidyl peptidase IX) were expressed and purified as described previously [54,55]. DPP4 (dipeptidyl peptidase IV) was purified from human seminal plasma as described previously [56]. Human recombinant PREP (prolylendopeptidase) was expressed and purified as described before [54].

*IC_50_* measurements for FAP and PREP were carried out as described before [30,55]. Briefly, Z-Gly-Pro-7-amino-4-methylcoumarine (AMC, Bachem) at a final concentration of 50 µM at pH 8.0 in 0.05 M Tris-HCl buffer, 1 mg/mL BSA and 140 mM NaCl was used as the substrate for FAP IC_50_ measurements. For PREP, N-succinyl-Gly-Pro-AMC (Bachem) at a final concentration of 250 µM at pH 7.4 in 0.1 M K-phosphate, 1 mM EDTA, 1 mM DTT and 1 mg/mL BSA buffer was used.

*IC_50_* measurements for DPP4, DPP8 and DPP9 were performed as published before, using Ala-Pro-pNA as the substrate at the respective concentrations of 25 µM (DPP4), 300 µM (DPP8) and 150 µM (DPP9) at pH 7.4 in 0.05 M HEPES-NaOH buffer with 0.1% Tween-20, 0.1 mg/mL BSA and 150 mM NaCl) [30,57].

All enzymatic activities were determined kinetically for 15 min at 37 °C by measuring initial velocities of AMC release (λex = 380 nm and λem = 465 nm) or pNA release (405 nm) from the substrates mentioned above with an Infinite 200 plate reader from Tecan Group Ltd. (Männedorf, Switzerland). The Magellan software was used to process the data, and data fitting was performed using a non-linear fitting model in Grafit 7.4. All *IC_50_* measurements were carried out in triplicate with at least eight different inhibitor concentrations tested.

### 2.5. Patient Study

#### 2.5.1. Radiosynthesis

[^68^Ga]Ga-DOTA.SA.FAPi was synthesized as described by Ballal et al. [58]. [^177^Lu]Lu-DOTAGA.(SA.FAPi)_2_ was synthesized as described by Ballal et al. [39].

[^177^Lu]Lu-DOTAGA.Glu.(FAPi)_2_ was radiolabeled by adding 6.1 GBq [^177^Lu]LuCl_3_ (obtained from BRIT (India) in 0.01 M supra pure HCl) in 0.1 M sodium acetate buffer (pH = 5.0) followed by 150 nmol DOTAGA.Glu.(FAPi)_2_ **7** (≈25 nmol/GBq). The solution was heated at 95 °C for 30 min followed by purification through Sep-Pak C18 light cartridge and eluted with 1 mL EtOH/H_2_O (1:1). Radiochemical quality control was carried out using the instant thin-layer chromatography method with 0.1 M trisodiumcitrate buffer (pH = 4.0) as solvent. The product was administered with a radiochemical purity of 95%. The radiochemical yield was 90%.

#### 2.5.2. Clinical Image Acquisition and Analysis

The patient underwent PET scans with [^18^F]F-FDG and [^68^Ga]Ga-DOTA.SA.FAPi mean injected activities of 185 and 222 MBq, respectively. The scans were acquired within a month time interval with a GE Discovery 710* 128 Slice PET/CT Scanner (40 mm detector at 0.35 s rotation speed). The whole-body PET/CT scans were acquired one hour post-injection. An initial scout image was acquired first, followed by a CT scan, and PET acquired at 2 min per bed. The whole-body CT scan parameters included 300–350 mAs, 120 kVp, slice thickness of 5 mm, and pitch 1 was acquired. [^18^F]F-FDG and [^68^Ga]Ga-DOTA.SA.FAPi PET/CT scans were loaded simultaneously and co-registered using carina as an anatomical landmark registration technique. The scan interpretations were conducted by two experienced nuclear medicine physicians and reviewed by a third. ROIs were drawn according to the PET Response Criteria in Solid Tumors (PERCIST 1.0). For quantitative assessment of standardized uptake values (SUV). corrected for lean body mass, 3D auto-contour ROI at a 40% threshold of SULpeak technique was used.

Planar whole-body scans after administration of [^177^Lu]Lu-DOTAGA.(SA.FAPi)_2_ or [^177^Lu]Lu-DOTAGA.Glu.(FAPi)_2_ were performed on a Dual Head Gamma Camera (GE Discovery NM/CT 670) using a high-energy general purpose (HEGP) collimator. Scans were acquired in the lutetium-177 window at a 20% energy window with peaks at 208 and 113 keV. The matrix size for the whole-body scan was 256 × 1024. Anterior and posterior views were acquired by using both detectors.

The clinical study was approved by the Institute Ethics committee at All India Institute of Medical Sciences (IECPG-22/27.02.2020). All patients gave their written informed consent.

## 3. Results and Discussion

### 3.1. Organic Synthesis

FAPi-NH_2_ **1** was synthesized according to the procedure in the supporting information (cf. Figure S1).

The synthesis of DOTAGA.Glu.(FAPi)_2_ **7** and DO3A.Glu.(FAPi)_2_ **11** is shown in Figure 2 and was started from FAPi-NH_2_ 1 and Boc-Glu-OH **2**.

First, 1 was coupled twice to obtain Boc-Glu.(FAPi)_2_ **3**. Here, HOBt and EDC*HCl with DIPEA in DMF were found to be the best coupling conditions, and **1** needs to be used in slight excess (2.5–3.0 eq). The cleavage of the Boc-protective group can be carried out under very mild conditions with 4 M HCl in 1,4-dioxane (8.6 eq) and acetonitrile as solvent, alternatively using trifluoracetic acid (TFA). The following coupling of the FAPi dimer Glu.(FAPi)_2_ **4** with DOTA-tris(tert-butyl ester) **8** and DOTAGA(*^t^*Bu)_4_ **5** were both carried out with HATU and DIPEA in DMF. For DO3A, only small amounts of product could be found in LC-MS measurements, and no product could be isolated after column chromatography. Therefore, an alternative route was tried out. Compound **8** was first converted into DOTA(*^t^*Bu)_3_-NHS **9**. After coupling with **4** with DIPEA in DMF, the *tert*-butyl ester groups of the chelator were cleaved and following RP-HPLC purification yielded DO3A.Glu.(FAPi)_2_ **11** with 20% over four steps. DOTAGA.Glu.(FAPi)_2_ **7** could be obtained with similar yields.

The ^nat^Lu-complexes were obtained by adding 0.1 M LuCl_3_ solution (2.0 eq) to 1 M HEPES buffer (pH = 5.5) and the corresponding precursors **7** and **11**, respectively. Subsequent RP-HPLC purification gave the Lu-complexed derivatives **^nat^Lu-7** and **^nat^Lu-11**.

### 3.2. Radiosynthesis

#### 3.2.1. [^68^Ga]Ga-DOTAGA.Glu.(FAPi)_2_ (**^68^Ga-7**)

**Figure 3:**
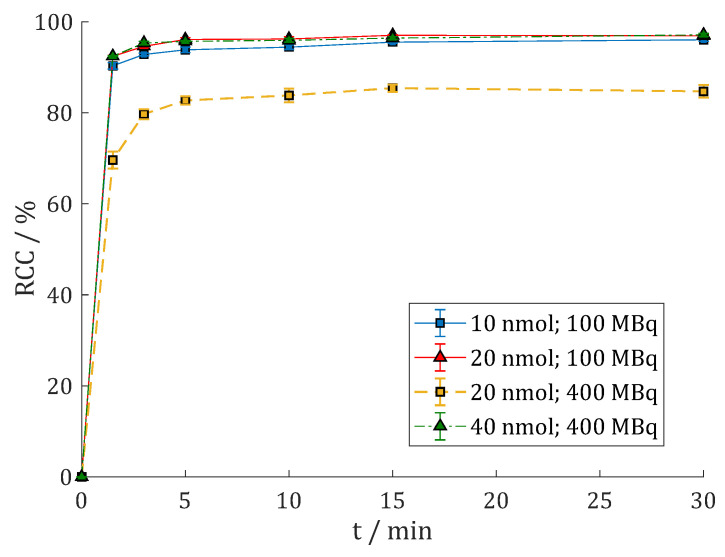
Reaction kinetics (radiochemical conversion (RCC) in %) of [^68^Ga]Ga-DOTAGA.Glu.(FAPi)_2_ **^68^Ga-7** in 1 M HEPES (pH = 4.5) at 95 °C with 100 MBq ^68^Ga (n = 4) or 400 MBq ^68^Ga (n = 2).

Moon et al. [31] observed that the ^68^Ga-radiolabeling of the dimers DOTA.(SA.FAPi)_2_ and DOTAGA.(SA.FAPi)_2_ reached better radiochemical conversion (RCC) rates with HEPES buffer compared to ammonium acetate (AmOAc) and sodium acetate (NaOAc). This could also be observed with the new dimers. Labeling of ca. 100 MBq ^68^Ga in 500 µL 1 M AmOAc (pH = 4.5) only gave 57% RCC with 10 nmol DOTAGA.Glu.(FAPi)_2_ **7** (cf. Figure S4). Furthermore, 20 nmol was needed to give good yields of >90%. In comparison, 10 nmol **7** and 100 MBq ^68^Ga in 500 µL 1 M HEPES (pH = 4.5) gave >90% after 1.5 min and >95% after 15 min (Figure 3). Similar results were achieved in a fourfold upscale with 400 MBq ^68^Ga. The second TLC system 1 M AmOAc/MeOH (1:1) (RCC = 98.8 ± 0.5%) and Radio-HPLC (RCP = 99.1%, cf. Figure S13) gave confirming results.

The complex stability was determined in human serum (HS) and phosphate-buffered saline (PBS) in triplicate each (n = 3) by incubating ca. 10 MBq of **^68^Ga-7** in 0.5 mL of HS/PBS at 37 °C for 120 min and analyzing via radio-TLC. The complex is stable under these conditions (Figure S5).

#### 3.2.2. [^177^Lu]Lu-DOTAGA.Glu.(FAPi)_2_ (**^177^Lu-7**)

**Figure 4:**
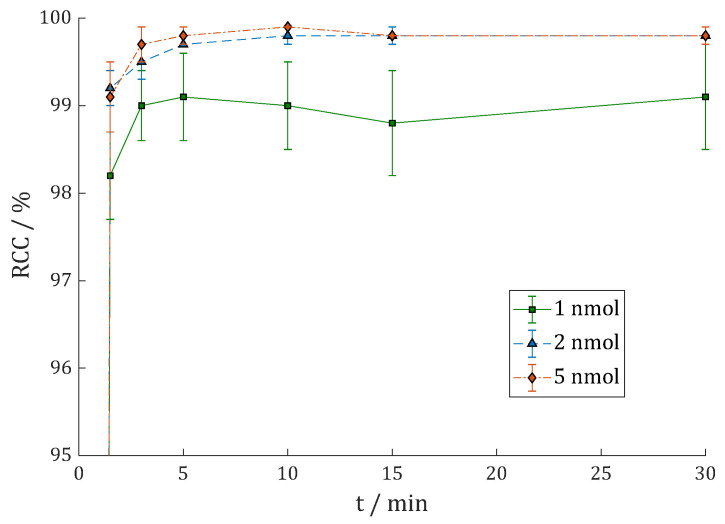
Reaction kinetics (radiochemical conversion (RCC) in %) of [^177^Lu]Lu-DOTAGA.Glu.(FAPi)_2_ **^177^Lu-7** in 1 M HEPES (pH = 5.5) at 95 °C with 50–100 MBq ^177^Lu (n = 3).

Labeling of **7** with lutetium-177 was carried out in 400 µL 1 M HEPES buffer (pH = 5.5) at 95 °C and gave excellent results with RCC > 99% (1 min) with 2 and 5 nmol **7** and >98% (1 nmol) with just 1 nmol that was given to the buffer solution with 50–100 MBq [^177^Lu]LuCl_3_ (Figure 4).

In a proportional upscale, this would lead to 10 nmol per GBq lutetium-177. The second radio-TLC system (RCC > 99%) and radio-HPLC (cf. Figure S14: RCP = 99.8%) also showed quantitative labeling without any subsequent purification step to separate free noncomplexed lutetium-177.

The complex stability of **^177^Lu-7** was determined similarly to the ^68^Ga-complex **^68^Ga-7**, except that the incubation at 37 °C was carried out for 14 days. **^177^Lu-7** showed no degradation, and the complex was stable over two half-lives (Figure S6).

#### 3.2.3. [^225^Ac]Ac-DOTAGA.Glu.(FAPi)_2_ (**^225^Ac-7**)

Actinium-225 (*t*_1/2_ = 9.9 d) is part of the neptunium cascade and has no dominant gamma-ray emission lines itself. Therefore, the imaging has to be performed via its daughter nuclides. The gamma lines of francium-221 (*t*_1/2_ = 4.9 min, Eγ = 218 keV) and bismuth-213 (*t*_1/2_ = 45.6 min, Eγ = 440 keV) are the predominant ones that can be used in TLC imaging.

The radio-TLCs were always imaged after one hour and one day. Waiting at least one hour is necessary since the equilibrium between actinium-225 and its daughters is disrupted while running the TLC, and the ^225^Ac/^221^Fr-equilibrium has to be reestablished. At this time point, bismuth-213 also contributed to the TLC imaging, which is why the ^225^Ac-RCY/RCC is systematically underestimated at this time point. This was broadly investigated with a statistic model by Kelly et al. [59] where they found that a RCP > 90% after 2 h results in a “true” RCP > 97% after 1 day. After one day, bismuth-213 and all the other daughter nuclides decayed. Imaging at this time point gives the distribution of actinium-225. In gamma spectroscopy the gamma-ray lines of francium-221 and bismuth-213 can be distinguished. The amount of actinium-225 can be calculated via the ^221^Fr peak; thus, waiting one hour is sufficient in this case.

At first, test labeling experiments were carried out. Therefore, 10 or 20 nmol of **7** were added to a solution of ca. 500 kBq [^225^Ac]AcCl_3_ and 0.1 M sodium ascorbate (pH = 7.0), which was heated at 95 °C for 60 min (n = 2). For 10 nmol, the RCC was 86.7 ± 3.6% (60 min, 1 d) when imaging after one day. As expected, the RCCs are slightly lower when imaging after one hour, in this case 84.3 ± 4.0% (60 min, 1 h). For 20 nmol, the RCCs were > 90% (15 min) and 91.8 ± 4.7% (60 min, 1 h) and 96.5 ± 1.9% (60 min, 1 d). Exemplary reaction kinetics are shown in Figure S5. It can be seen that at early imaging time points (e.g., 1 h), four spots can be seen, with R_f_ ≈ 0.5 being free actinium-225 and R_f_ = 0.0 being the ^225^Ac-complex [^225^Ac]Ac-DOTAGA.Glu.(FAPi)_2_ **^225^Ac-7**. They can also be seen when the exact same TLC is measured again the next day. The other two spots disappear at the radio-TLc after one day. When measuring at additional time points (e.g., 2 h, 3 h etc.), gradually decreasing intensity is observed. This most likely fits the physical half-life of ^213^Bi and the ability of Bi to form complexes with DOTA conjugates as a trivalent metal. Therefore, R_f_ > 0.7 is free ^213^Bi, whereas the spot at R_f_ = 0.1–0.2 is considered to be [^213^Bi]Bi-DOTAGA.Glu.(FAPi)_2_, also due its gradual increasing intensity during the reaction.

**Figure 5:**
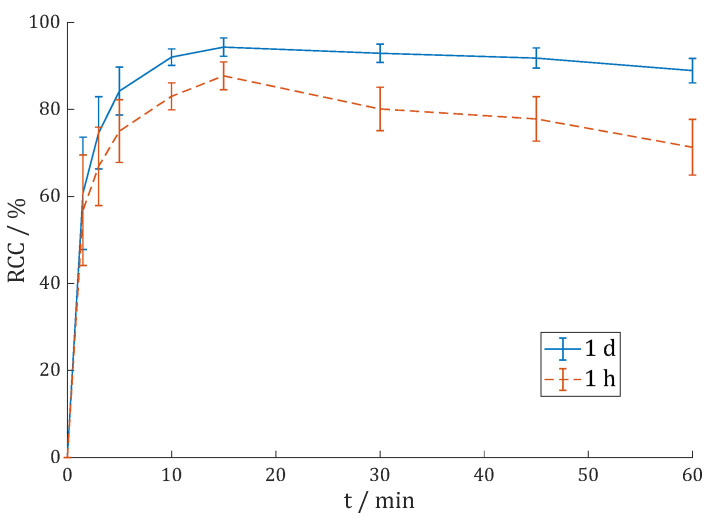
Reaction kinetics (radiochemical conversion (RCC) in %) of [^225^Ac]Ac-DOTAGA.Glu.(FAPi)_2_ **^225^Ac-7** in 0.1 M sodium ascorbate (pH = 7.0) at 95 °C with 1.6–3.2 MBq ^225^Ac (n = 3).

Higher amounts were used for labeling in similar conditions: 1.6–3.2 MBq [^225^Ac]AcCl_3_ and 30–40 nmol/MBq were heated in 1 mL 0.1 M sodium ascorbate (pH = 7.0) at 95 °C for 60 min (n = 3). Using 40 instead of 30 nmol/MBq did not significantly influence the RCC; hence, the data were merged. The reaction kinetics were analyzed via radio-TLCs (Figure 5) that were imaged one hour after the TLC had been developed, and the exact same TLC was measured again the next day.

Figure 5 shows the underestimation of the imaging after one hour compared to imaging after one day. The maximum RCC of 87.7 ± 3.2% (1 h) and 94.3 ± 2.1% (1 d) is reached after 15 min of heating. The RCC is not quantitative, but increasing to 40 nmol/MBq did not increase RCC. Higher activity concentrations per volume (MBq/mL) might improve the yield, for example by using more activity, less volume or both.

With longer heating times, the RCC decreases again, which might indicate radiolysis. The effect is stronger in the data after one hour. Kelly et al. [59] also found out that the underestimation is not linear and varies depending on the value of RCP/RCC, which might play a role here. In conclusion, the heating should be shortened (15–30 min) in future ^225^Ac-labelings of **7**. Lower reaction temperatures should also be investigated in future labeling experiments since the solution became slightly yellow after long heating times, which is probably due to the oxidation of ascorbate.

The RCCs were determined with AmOAc (pH = 4.0)/MeOH (1:1) radio-TLC and gamma spectroscopy measurements of a citrate TLC strip match and confirm the results (Table 1).

For the complex stability measurements of **^225^Ac-7** in HS and PBS, 350–400 kBq of the labeling solution was directly added to HS and PBS (0.5 mL, n = 3) and incubated for 20 days at 37 °C (Figure S9). The complex [^225^Ac]Ac-DOTAGA.Glu.(FAPi)_2_ remains stable in PBS for a couple of days (89.9 ± 0.7% after 2 d), but then, gradual radiolysis occurs (45.7 ± 0.2% after 20 d). In contrast, the stability in HS is much higher despite having the same activity concentration (85.5 ± 1.7% after 20 d). Higher dilution and the addition of sodium ascorbate and ethanol results in a high stability of the pure final formulation (95.4 ± 1.0% after 20 d). Both are known to be scavengers and are able to reduce radiolysis. It seems reasonable to dilute the final product and add scavengers as much as possible, especially for ^225^Ac-radiopharmaceuticals due to the high-energy alpha-particles and the high potential for radiolysis.

#### 3.2.4. [^68^Ga]Ga-DO3A.Glu.(FAPi)_2_ (**^68^Ga-11**)

**Figure 6:**
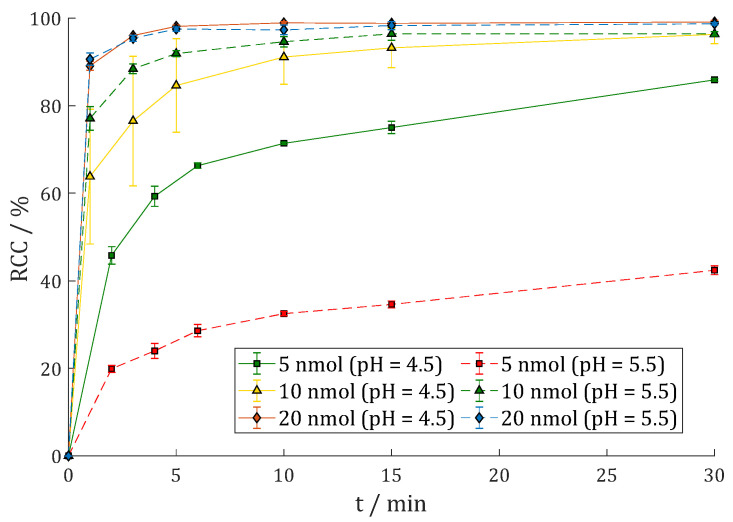
Reaction kinetics (radiochemical conversion (RCC) in %) of [^68^Ga]Ga-DO3A.Glu.(FAPi)_2_ **^68^Ga-11** in 1 M HEPES (pH = 4.5 or 5.5) at 95 °C with 100 MBq ^68^Ga (n = 3).

Labeling of DO3A.Glu.(FAPi)_2_ **11** with gallium-68 was performed similarly to labeling of **7**. Since the generator had much lower activities, cationic post-processing was carried out before labeling [53]. In addition, 1 M HEPES buffer was tested at two different pH values (4.5 and 5.5). Three different precursor amounts were used to label 100 MBq ^68^Ga at each pH value (Figure 6).

Only with 5 nmol of precursor **11** the differences were significant, and pH = 4.5 seems to be better suited than pH = 5.5 (85.9 ± 0.3% vs. 42.4 ± 1.0% after 30 min). For 10 nmol (96.3 ± 2.1% vs. 96.4 ± 0.5%) and 20 nmol (99.1 ± 0.4% vs. 98.7 ± 0.3%), the RCCs were almost identical. This suggests that the pH value of the reaction is flexible to a certain extent. Radio-HPLC (Figure S15) shows RCP = 98.7%.

The complex stability of **^68^Ga-11** was determined analogously to **^68^Ga-7**. Almost no radiolysis had taken place, and after 120 min, still > 98% of complexes were intact in both HS and PBS (Figure S10).

#### 3.2.5. [^177^Lu]Lu-DO3A.Glu.(FAPi)_2_ (**^177^Lu-11**)

**Figure 7:**
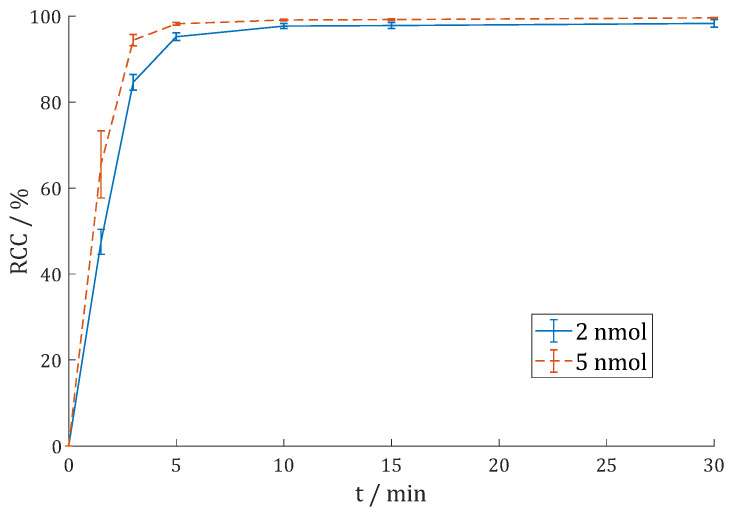
Reaction kinetics (radiochemical conversion (RCC) in %) of [^177^Lu]Lu-DO3A.Glu.(FAPi)_2_ **^177^Lu-11** in 1 M HEPES (pH = 5.5) at 95 °C with 50–100 MBq ^177^Lu (n = 5).

[^177^Lu]Lu-DO3A.Glu.(FAPi)_2_ **^177^Lu-11** was synthesized similarly to the DOTAGA derivative **^177^Lu-7**. When comparing the reaction kinetics in Figure 7 with Figure 4, the labeling kinetics of **^177^Lu-11** are somewhat slower than those of **^177^Lu-7**.

Whereas the RCC is >99% after 1.5 min for 2 nmol **13**, it takes 5 min to reach >95% with **^177^Lu-11**, and even 5 min to obtain RCC > 98% with 5 nmol **^177^Lu-11**. Nevertheless, **^177^Lu-11** can be synthesized in practically quantitative RCCs (>98% with 2 nmol for 30 min; >99% with 5 nmol for 10 min). The second TLC system and radio-HPLC (cf. Figure S16) also gave almost quantitative RCC/RCP.

The complex stability measurements of **^177^Lu-11** were performed as described before for **^177^Lu-7**. Slight decomposition could be observed in PBS (94.7 ± 2.0% intact conjugates after 14 days), whereas **^177^Lu-11** seems to be stable in HS, with both having 20 MBq/mL in the beginning (Figure S11).

**^177^Lu-11** is slightly less stable than **^177^Lu-7** since the additional carboxyl group of DOTAGA might be able to take part in the complexation of lutetium-177, which could give it additional stability. Lutetium can form a complex with unconjugated DOTA where all eight donor ligands (4 × N in the macrocyclic + 4 × O^−^ from the acetate arms) are part of the complex [60].

#### 3.2.6. [^90^Y]Y-DO3A.Glu.(FAPi)_2_ (**^90^Y-11**)

**Figure 8:**
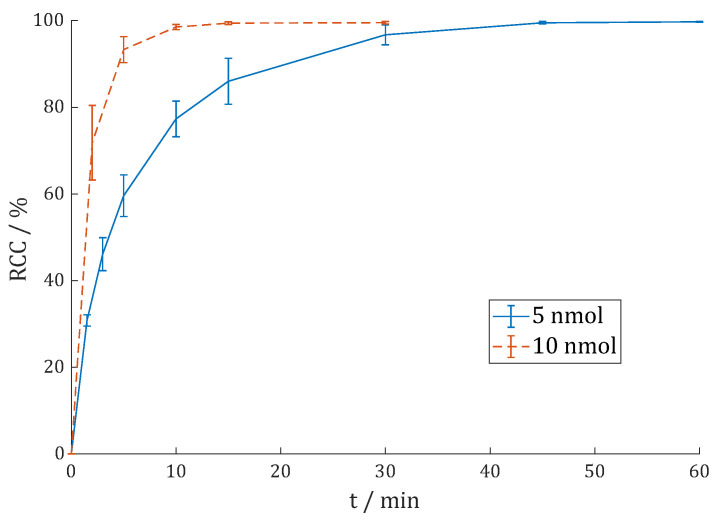
Reaction kinetics (radiochemical conversion (RCC) in %) of [^90^Y]Y-DO3A.Glu.(FAPi)_2_ **^90^Y-11** in 1 M HEPES (pH = 4.5) at 95 °C with 30–60 MBq ^90^Y (n = 3).

DO3A.Glu.(FAPi)_2_ **11** was additionally labeled with yttrium-90 in 1 M HEPES buffer (pH = 4.5). For these reaction kinetic studies, 5 and 10 nmol **11** were labeled with 30–60 MBq [^90^Y]YCl_3_ in 400 µL buffer at 95 °C. The reaction kinetics were analyzed via radio-TLC and (see Figure 8).

Quantitative RCC > 99% could be achieved with 10 nmol in 15 min. With 5 nmol, the reaction is much slower, but after 45 min, RCC > 99% could also be reached.

Subsequent complex stability studies of **^90^Y-11** were carried out the same way as with ^68^Ga- and ^177^Lu-cmplexes. The complex can be considered stable (>98%) in both HS and PBS after more than two half-lives (6 days) at 37 °C (see Figure S12).

#### 3.2.7. Lipophilicity

The lipophilicity was investigated experimentally via the shake-flask method for the ^68^Ga-complexes **^68^Ga-7** and **^68^Ga-11** as well as for the ^177^Lu-complexes **^177^Lu-7** and **^177^Lu-11** with ca. 10 MBq per vial (n = 4) with PBS and 1-octanol. The log*D*_7.4_ values were obtained by determining the integral of each spot on the radio-TLC and calculation with Equation (1). Table 2 gives an overview and compares the log*D*_7.4_ values with those of previous works from our group [29,31].

#### 3.2.8. Comparison with DOTAGA.(SA.FAPi)_2_

The monomeric radiotracer [^68^Ga]Ga-DOTA.SA.FAPi showed relatively strong hydrophilic characteristics. This may be one of the reasons that in vivo PET studies indicated a relatively fast renal clearance [58]. In contrast, the dimer [^177^Lu]Lu-DOTAGA.(SA.FAPi)_2_ predominantly showed biliary excretion over renal excretion [39]. The washout from the hepatobiliary system/liver was slower than that of the monomeric radiotracers (the same for the renal excretion). In particular, [^177^Lu]Lu-DOTAGA.(SA.FAPi)_2_ remained in the large intestines/colon region for a longer time during excretion. The colon was identified as the critical organ, and the radiation dose to the kidneys was increased compared to the monomer [39,52]. Therefore, we tried to synthesize compounds that are more hydrophilic than DOTAGA.(SA.FAPi)_2_ to reduce biliary excretion and thus the radiation dose to the colon.

The DOTAGA complexes **^68^Ga-7** and **^177^Lu-7** are definitely more hydrophilic than the first-generation dimer DOTAGA.(SA.FAPi)_2_ and the DOTA complexes **^68^Ga-11** and **^177^Lu-11**. The hydrophilicity of **^68^Ga-7** is comparable to the monomer [^68^Ga]Ga-DOTA.SA.FAPi. Radioligand therapy (RLT) with radiometals such as lutetium-177 is the main objective of these new dimers. [^177^Lu]Lu-DOTAGA.Glu.(FAPi)_2_ **^177^Lu-7** shows the highest hydrophilicity of all complexes (log*D_7._*_4_ = −2.77 ± 0.10). There is no log*D*_7.4_ value for [^177^Lu]Lu-DOTA.SA.FAPi that allows for a direct comparison. Nonetheless, these results are very promising to translate this molecule from bench to bedside.

The molecular design of the FAPi homodimers DOTA.(SA.FAPi)_2_ and DOTAGA.(SA.FAPi)_2_ may create radiolabeling limitations due to steric hindrance as a result of the chelator being in the center of the molecule. The DOTA chelator only has six donor ligands because it is attached to the two targeting vectors on two binding sites, which only allow for labeling with gallium-68 [31]. The DOTAGA derivative has seven donor ligands for complexation and can be labeled with lutetium-177, while it was cumbersome in the case of actinium-225. The new FAPi dimer DOTAGA.Glu.(FAPi)_2_ **7** has eight donor ligands and can be labeled with actinium-225 with high RCC > 90%. ^225^Ac-labeling of DO3A.Glu.(FAPi)_2_ **11** was just as problematic as with DOTAGA.(SA.FAPi)_2_. In general, DOTA-conjugated precursors have been investigated for ^225^Ac-labeling quite extensively and allow for efficient labeling when the chelator is in exo-position [61,62,63,64,65,66].

In summary, DOTAGA.Glu.(FAPi)_2_ showed superior labeling properties compared to the other dimeric compounds.

### 3.3. In Vitro Inhibition Assays

High affinity for FAP and low affinity for the very similar proteases PREP and DPPs (DPP4, DPP8 and DPP9) are important requirements for FAPi-based radiopharmaceuticals to be promising candidates for subsequent in vivo studies.

The IC_50_ values were determined in in vitro studies. Table 3 summarizes the results of the IC_50_ measurements. The selectivity index (SI) is the ratio of the IC_50_ value of the respective protease (PREP, DPP4, DPP8 and DPP9) to the IC_50_ value of FAP.

Compounds **7**, **^nat^Lu-7** and **11** show very high affinity for FAP, as reflected in the subnanomolar IC_50_ values. The affinities of **7** and **^nat^Lu-7** are very similar, and the different selectivity indexes do not differ substantially as well. This supports previous results where the IC_50_ values of the precursor and its respective metal complex (^nat^Ga or ^nat^Lu) do not differ much [29,30,31], as can be seen with DOTAGA.(SA.FAPi)_2_ and [^nat^Lu]Lu-DOTAGA.(SA.FAPi)_2_ for example (see Table 3) [31]. The affinity and selectivity for all new compounds were better than for DOTAGA.(SA.FAPi)_2_. This also includes the comparison of the Lu-complex **^nat^Lu-7** with [^nat^Lu]Lu-DOTAGA.(SA.FAPi)_2_.

Although selectivity indexes (SIs) are lower compared to UAMC-1110, high selectivity for FAP over PREP and DPP4 was measured, which are most relevant among the related proteases. In contrast to FAP, they are ubiquitously expressed in healthy tissue. A high affinity for PREP or DPP4 would lead to worsened tumor selectivity and a diminished tumor-to-background ratio in PET/SPECT scans and/or RNT. Overall, these results strongly suggest that compounds **7** and **11** might also have superior properties in vivo compared to DOTAGA.(SA.FAPi)_2_ and that especially FAPi dimer **7** is suitable for further investigations.

The IC_50_(FAP) values reported for BiOncoFAP-DOTAGA and [^nat^Lu]Lu-BiOncoFAP-DOTAGA were 0.17 and 0.19 nM, respectively [50]. These values seem to be equal to **7** and **^nat^Lu-7**, although it has to be noted that the assay conditions were different (substrate/enzyme/buffer concentration, temperature, volume etc.). For the dimer [^68^Ga]Ga-DOTA-2P(FAPI)_2_, the assay setup differed drastically, as a competitive assay of the radiolabeled agent against FAPI-46 was carried out and resulted in an IC_50_ = 3.68 ± 1.82 nM [48]. The different assay conditions, especially of Zhang et al. [48], limit the comparability of the different IC_50_ values. On top, no IC_50_ values for the related proteases and therefore no selectivity indexes were reported by Zhao et al. [48] or by Galbiati et al. [50].

### 3.4. Patient Study (Medullary Thyroid Cancer)

A 40-year-old male was diagnosed with medullary thyroid cancer in 2016. The patient underwent total thyroidectomy and bilateral radical neck dissection in prior treatments.

**Figure 9:**
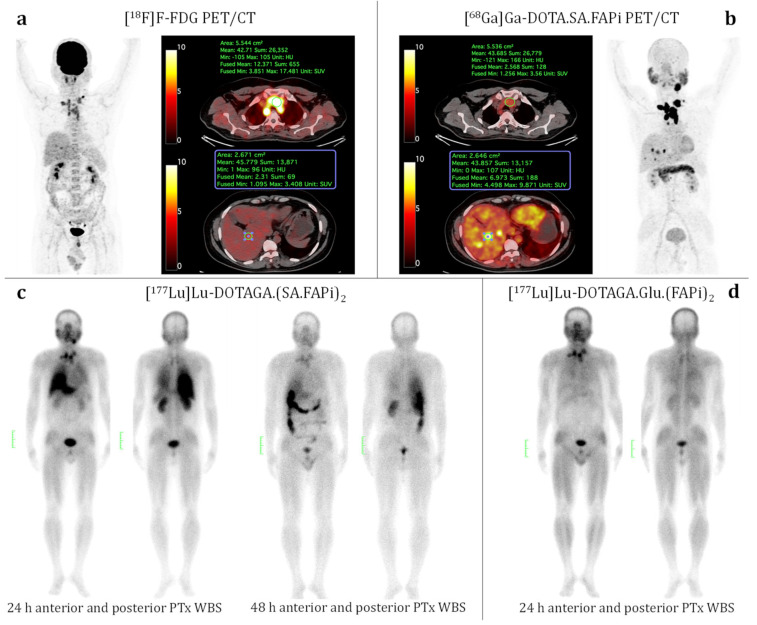
A 40-year-old male diagnosed with medullary thyroid carcinoma underwent [^18^F]F-FDG PET/CT scan (**a**) followed by [^68^Ga]Ga-DOTA.SA.FAPi PET/CT scan (**b**) with the scale being SUV in %ID/g. The patient underwent two cycles of 5.5 GBq of [^177^Lu]Lu-DOTAGA.(SA.FAPi)_2_ therapy (**c**). The post-treatment whole-body scan at 24 h p.i. (**c**, **left**) reveals intense uptake in the neck and mediastinal metastases but a simultaneous intense radiotracer accumulation in the liver. In addition, 48 h p.i. shows the hepatobiliary excretion (**c**, **right**). Subsequent treatment in the same patient with 5.5 GBq of the new dimer [^177^Lu]Lu-DOTAGA.Glu.(FAPi)_2_ **^177^Lu-7** showed only uptake in the neck lesions on whole-body scan at 24 h p.i. (**d**) and negligible uptake in the liver.

[^18^F]F-FDG and [^68^Ga]Ga-DOTA.SA.FAPi PET/CT scans were carried out. [^18^F]F-FDG PET/CT (see Figure 9a) showed an FDG-avid soft tissue lesion noted measuring 3 × 3.5 cm in the thyroid bed region (SULpeak: 3.4), avid bilateral level VI, bilateral supraclavicular lymph nodes noted, sub-centimetric tracer avid level V lymph nodes, superior mediastinal/bilateral upper paratracheal, and bilateral paraoesophageal lymph nodes. Few hypodense lesions were noted in the right lobe of the liver involving segment V with low [^18^F]F-FDG uptake of 1.75. Diffuse physiological FDG distribution was seen in the axial and appendicular skeleton. Unlike [^18^F]F-FDG, [^68^Ga]-DOTA.SA.FAPi showed superior uptake in all the lesions (soft tissue neck lesion: SULpeak 17.98) corresponding to [^18^F]F-FDG PET/CT scan and better delianiation in the liver lesions (SULpeak:10.5) on MIP itself (see Figure 9b). The patient was administered two cycles of [^177^Lu]Lu-DOTAGA.(SA.FAPi)_2_. Figure 9c shows whole-body scans at 24 and 48 h post-injection. Despite uptake in the lesions (neck nodes and mass), huge amounts of radiotracer uptake were observed in the liver (24 h p.i.) and colon (48 h p.i.), causing high radiation burden to the colon and liver. For the third cycle of treatment, the new FAPi dimer [^177^Lu]Lu-DOTAGA.Glu.(FAPi)_2_ **^177^Lu-7** was considered.

The patient was administered 5.5 GBq of [^177^Lu]Lu-DOTAGA.Glu.(FAPi)_2_ **^177^Lu-7,** which was previously synthesized according to the procedure described in Methods and Materials (Section 2.5.1). Furthermore, 24 h p.i. high uptake in the neck and mediastinal metastases was comparable to the first-generation dimer. In contrast, only negligible radiotracer uptake in the liver and colon was observed. This suggests a different excretion pattern, possibly a higher proportion of renal instead of hepatobiliary excretion (improved pharmacodynamic profile) or a faster washout (improved pharmacokinetic profile), or both, which results in a significantly reduced radiation dose to the critical healthy organs liver and colon.

While this is a first proof-of-concept investigation and not of quantitative dimension, the results support the assumption that the new ^177^Lu-labeled FAPi homodimer **^177^Lu-7** has favorable pharmacologic properties compared to [^177^Lu]Lu-DOTAGA.(SA.FAPi)_2_. More detailed studies regarding biodistribution as well as safety and efficacy are currently ongoing, which are needed to better understand the pharmacokinetic and pharmacodynamic behavior of [^177^Lu]Lu-DOTAGA.Glu.(FAPi)_2_ **^177^Lu-7** in systematic studies.

## 4. Conclusions

DOTAGA.Glu.(FAPi)_2_ and DO3A.Glu.(FAPi)_2_ represent the second generation of our homodimeric FAPi compounds. They were synthesized with glutamic acid as a central linker unit and successfully labeled with different trivalent radiometals. Both precursors show excellent labeling properties with ^68^Ga and ^177^Lu. In contrast to DO3A.Glu.(FAPi)_2_ and the first-generation dimer DOTAGA.(SA.FAPi)_2_, DOTAGA.Glu.(FAPi)_2_ could also be labeled successfully with ^225^Ac in high yields. All complexes showed high stability and hydrophilicity. Beyond that, IC_50_ values were determined to confirm the very high affinity for fibroblast activation protein alpha (FAP) and high selectivity for FAP over prolyl endopeptidase (PREP) and the dipeptidyl peptidase (DPP4). The in vitro studies showed the best results for DOTAGA.Glu.(FAPi)_2_, but the results for DO3A.Glu.(FAPi)_2_ were also better than for DOTAGA.(SA.FAPi)_2_. It is also noteworthy that the respective FAP affinity of the new homodimeric compounds is higher than for the initial FAP inhibitor UAMC-1110 itself, which was measured in the same assay. Similar FAP affinities were reported for BiOncoFAP-DOTAGA and its ^nat^Lu-complex by Galbiati et al. [50], but no selectivity data were reported. The comparison of the IC_50_ values is difficult and should be cautiously interpreted, as the inhibition assays are different from each other (especially regarding Zhao et al. [48]).

DOTAGA.Glu.(FAPi)_2_ proved to be the most promising candidate for further in vivo studies that are currently ongoing. The compound seems especially suited for therapeutic applications with ^177^Lu, ^90^Y and ^225^Ac. Especially, FAP-targeted alpha-particle therapy (FAP-TAT) with ^225^Ac-radiopharmaceuticals is a new treatment option that could potentially be more efficacious than with beta-emitting therapeutics since stromal cells, similar to how CAFs are reported to be more resistant to radiation [67,68,69]. Therefore, particles with higher energy and linear energy transfer such as alpha particles might be needed to address the specifics of the tumor stroma. The stroma can build a physical barrier around the tumor that is hard to penetrate for some agents (e.g., chemotherapeutics), which results in poor efficacy [5,70,71]. The destruction of tumor stroma with alpha-emitters in combination with other non-radioactive agents (e.g., chemotherapeutics, targeted drug delivery systems, CAR T-cell therapy etc.) might also be a promising therapy approach, and [^225^Ac]Ac-DOTAGA.Glu.(FAPi)_2_ might be an interesting candidate for FAP-TAT in the future.

A first proof-of-concept patient study with [^177^Lu]Lu-DOTAGA.Glu.(FAPi)_2_ showed promising results. The pharmacological properties are superior to those of [^177^Lu]Lu-DOTAGA.(SA.FAPi)_2_. with a high radiotracer uptake and long tumor retention in lesions. At the same time, the excretion kinetics are faster, and minimized uptake in non-target tissues, especially the colon as the critical organ for [^177^Lu]Lu-DOTAGA.(SA.FAPi)_2_, was observed. Improved pharmacologic properties are essential to ensure a safe therapy without adverse side effects. Further studies are currently ongoing and will investigate biodistribution, efficacy and safety in more detail.

## Figures and Tables

**Table 1:** Comparison of the determined radiochemical conversion rates (RCCs) with the different methods at different time points of the reaction (15 and 60 min: n = 3; after C18 cartridge purification: n = 1). The imaging/measurement was carried out 1 hour (1 h) or 1 day (1 d) after the radio TLC had been developed.

RCC/%	1 h (Citrate)	1 d (Citrate)	1 d (AmOAc/MeOH)	1 h (γ)
after 15 min	87.7 ± 3.2%	94.3 ± 2.1%	-	-
after 60 min	71.3 ± 6.4%	88.9 ± 2.8%	90.8 ± 2.3%	94.8 ± 2.5%
after C18	93.9%	98.1%	97.9%	100%

**Table 2:** log*D*_7.4_ values of different labeled FAPi-based radiopharmaceuticals. The values for [^68^Ga]Ga-DOTA.SA.FAPi and [^68^Ga]Ga-DOTAGA.(SA.FAPi)_2_ are taken from Moon et al. [29] and Moon et al. [31].

Complex	log*D_7._*_4_
[^68^Ga]Ga-DOTA.SA.FAPi [29]	−2.68 ± 0.06
[^68^Ga]Ga-DOTAGA.(SA.FAPi)_2_ [31]	−2.02 ± 0.06
[^68^Ga]Ga-DOTAGA.Glu.(FAPi)_2_ (**^68^Ga-7**)	−2.48 ± 0.05
[^177^Lu]Lu-DOTAGA.Glu.(FAPi)_2_ (**^177^Lu-7**)	−2.77 ± 0.10
[^68^Ga]Ga-DO3A.Glu.(FAPi)_2_ (**^68^Ga-11**)	−2.08 ± 0.07
[^177^Lu]Lu-DO3A.Glu.(FAPi)_2_ (**^177^Lu-11**)	−1.77 ± 0.10

**Table 3:** IC_50_ value and selectivity index (SI) of different FAP inhibitors: DOTAGA.Glu.(FAPi)_2_ **7**, [^nat^Lu]Lu-DOTAGA.Glu.(FAPi)_2_ **^nat^Lu-7**, DO3A.Glu.(FAPi)_2_ **11** as well as DOTAGA.(SA.FAPi)_2_, [^nat^Lu]Lu-DOTAGA.(SA.FAPi)_2_ and the initial FAP inhibitor UAMC-1110 from previous works (Jansen et al. [24] and Moon et al. [31]).

Compound	DOTAGA.Glu.(FAPi)_2_ (7)	[^nat^Lu]Lu-DOTAGA.Glu.(FAPi)_2_ (^nat^Lu-7)	DO3A.Glu.(FAPi)_2_ (11)	DOTAGA.(SA.FAPi)_2_ [31]	[^nat^Lu]Lu- DOTAGA.(SA.FAPi)_2_ [31]	UAMC-1110 [24,31]
*IC_50_(FAP)*/nM	0.26 ± 0.04	0.33 ± 0.02	0.60 ± 0.04	0.92 ± 0.06	1.54 ± 0.15	0.43 ± 0.02
*IC_50_(PREP)*/µM	0.59 ± 0.10	0.43 ± 0.16	1.00 ± 0.14	0.39 ± 0.02	0.56 ± 0.04	1.80 ± 0.01
*IC_50_(DPP4)/*µM	1.19 ± 0.08	0.65 ± 0.04	0.54 ± 0.06	0.40 ± 0.07	0.63 ± 0.07	>10
*IC_50_(DPP8)/*µM	0.029 ± 0.004	0.22 ± 0.02	1.03 ± 0.18	0.42 ± 0.04	0.41 ± 0.03	>10
*IC_50_(DPP9)/*µM	0.083 ± 0.0015	0.19 ± 0.01	0.95 ± 0.11	0.16 ± 0.02	0.18 ± 0.02	4.70 ± 0.40
*SI*(*PREP/FAP)*	2269	1292	1667	424	364	4186
*SI(DPP4/FAP)*	4577	1967	900	435	409	23,256
*SI(DPP8/FAP)*	112	661	1717	456	266	23,256
*SI(DPP9/FAP)*	319	557	1583	174	117	10,930

## Data Availability

Data are contained within the article and Supplementary Material.

## Supplementary Materials

The following supporting information can be downloaded at: https://www.mdpi.com/article/10.3390/cancers15061889/s1, Figure S1: Organic synthesis of FAPi-NH_2_ **1**: (a) HBr (47%), 126 °C, 4 h, 96%; (b) Boc_2_O, TEA, THF, RT, 19 h, 66%; (c) HBr (47%), 126 °C, 1 d, 100%; (d) SOCl_2_, MeOH, 0 °C–RT, 2 d, 100%; (e) Cs_2_CO_3_, DMF, 70 °C, 1 d, 59%; (f) 1 M LiOH, 1,4-dioxane, RT, 4 h, 57%; (g) HATU, DIPEA, DCM/DMF (1:1), RT, 19 h, 86%; (h) TFAA, pyridine, THF, DCM, RT, 3 h, 81%; (i) TFA, MeCN, RT, 5 h, h, 100%; (j) HBTU, HOBt, DIPEA, DMF, RT, 1 d, 72%; (k) 4 M HCl in 1,4-dioxane, MeCN, 0 °C–RT, 7 h, 100%; Figure S2: Analytical RP-HPLC of DOTAGA.Glu.(FAPi)_2_ **7**, 20–30% MeCN + 0.1% TFA (linear gradient) in 20 min, 5 mL/min: purity = 99.0%; Figure S3: Analytical RP-HPLC of DO3A.Glu.(FAPi)_2_ **11**, 20–30% MeCN + 0.1% TFA (linear gradient) in 20 min, 5 mL/min: purity = 98.5%; Figure S4: Reaction kinetics (radiochemical conversion (RCC) in %) of [^68^Ga]Ga-DOTAGA.Glu.(FAPi)_2_ **^68^Ga-7** in 1 M AmOAc (pH = 4.5) at 95 °C with 100 MBq ^68^Ga (n = 1); Figure S5: Complex stability of [^68^Ga]Ga-DOTAGA.Glu.(FAPi)_2_ **^68^Ga-7** in human serum (HS) and phosphate-buffered saline (PBS) over 120 min (n = 3); Figure S6: Complex stability of [^177^Lu]Lu-DOTAGA.Glu.(FAPi)_2_ **^177^Lu-7** in human serum (HS) and phosphate-buffered saline (PBS) over 14 days (n = 3); Figure S7: Exemplary reaction kinetic of [^225^Ac]Ac-DOTAGA.Glu.(FAPi)_2_ **^225^Ac-7** at 95 °C analyzed with 0.1 M citrate buffer (pH = 4.0) radio-TLC from 0–15 min (left to right). Exemplary profiles of citrate radio-TLC after 60 minutes at 95 °C are shown in the middle. The corresponding 1 M AmOAc (pH = 4.0)/MeOH (1:1) radio-TLCs are shown on the right. All TLCs were imaged at different time points after 1 h (top) and 1 day (bottom). These measurements together make clear that: R_f_(^225^Ac-complex=product **^225^Ac-7**) = 0.0 and R_f_(free ^225^Ac) ≈ 0.5. The other two spots appearing in a) must be from one of the short-lived daughter nuclides, most likely ^213^Bi since the spots are slowly decaying when measuring the TLC over several hours, which fits the physical half-life and Bi is a trivalent metal that can be complexed by DOTA conjugates. Therefore, R_f_(free ^213^Bi) > 0.7 can be assigned to be free ^213^Bi and R_f_(^213^Bi-complex) = 0.1–0.2 is considered to be the ^213^Bi-complex; Figure S8: Reaction kinetics (radiochemical conversion (RCC) in %) of [^225^Ac]Ac-DOTAGA.Glu.(FAPi)_2_ **^225^Ac-7** in 0.1 M sodium ascorbate (pH = 7.0) at 95 °C with 500 kBq ^225^Ac (n = 2); Figure S9: Complex stability of [^225^Ac]Ac-DOTAGA.Glu.(FAPi)_2_ **^225^Ac-7** in human serum (HS) and phosphate-buffered saline (PBS) (0.7–0.8 MBq/mL) and final formulation (“pure”, 0.2 MBq/mL) after C18 cartridge purification over 20 days (n = 3); Figure S10: Complex stability of [^68^Ga]Ga-DO3A.Glu.(FAPi)_2_ **^68^Ga-11** in human serum (HS) and phosphate-buffered saline (PBS) over 120 min (n = 3); Figure S11: Complex stability of [^177^Lu]Lu-DO3A.Glu.(FAPi)_2_ **^177^Lu-11** in human serum (HS) and phosphate-buffered saline (PBS) over 14 days (n = 3); Figure S12: Complex stability of [^90^Y]Y-DO3A.Glu.(FAPi)_2_ **^90^Y-11** in human serum (HS) and phosphate-buffered saline (PBS) over 6 days (n = 3); Figure S13: Analytical Radio-HPLC of [^68^Ga]Ga-DOTAGA.Glu.(FAPi)_2_ **^68^Ga-7** (*t_R_* = 10.5 min, 40 nmol **7**, 400 MBq ^68^Ga), 20–70% MeCN + 0.1% TFA (linear gradient) in 20 min: RCP = 99.1%; Figure S14: Analytical Radio-HPLC of [^177^Lu]Lu-DOTAGA.Glu.(FAPi)_2_ **^177^Lu-7** (*t_R_* = 10.3 min, 1 nmol **7**, 100 MBq ^177^Lu), 20–50% MeCN + 0.1% TFA (linear gradient) in 20 min: RCP = 99.8%; Figure S15: Analytical Radio-HPLC of [^68^Ga]Ga-DO3A.Glu.(FAPi)_2_ **^68^Ga-11** (*t_R_* = 9.5 min, 20 nmol **11**, 100 MBq ^68^Ga), 10–90% MeCN + 0.1% TFA (linear gradient) in 20 min: RCP = 98.7%; Figure S16: Analytical Radio-HPLC of [^177^Lu]Lu-DO3A.Glu.(FAPi)_2_ **^177^Lu-11** (*t_R_* = 10.1 min, 2 nmol, 100 MBq ^177^Lu), 20–50% MeCN + 0.1% TFA (linear gradient) in 10 min: RCP = 99.9%.

## References

[1] O’Brien P., O’Connor B.F. (2008). Seprase: An Overview of an Important Matrix Serine Protease. Biochim. Biophys. Acta—Proteins Proteom..

[2] Rettig W.J., Garin-Chesa P., Beresford H.R., Oettgen H.F., Melamed M.R., Old L.J. (1988). Cell-Surface Glycoproteins of Human Sarcomas: Differential Expression in Normal and Malignant Tissues and Cultured Cells. Proc. Natl. Acad. Sci. USA.

[3] Milner J.M., Kevorkian L., Young D.A., Jones D., Wait R., Donell S.T., Barksby E., Patterson A.M., Middleton J., Cravatt B.F. (2006). Fibroblast Activation Protein Alpha Is Expressed by Chondrocytes Following a Pro-Inflammatory Stimulus and Is Elevated in Osteoarthritis. Arthritis Res. Ther..

[4] Levy M.T., McCaughan G.W., Abbott C.A., Park J.E., Cunningham A.M., Müller E., Rettig W.J., Gorrell M.D. (1999). Fibroblast Activation Protein: A Cell Surface Dipeptidyl Peptidase and Gelatinase Expressed by Stellate Cells at the Tissue Remodelling Interface in Human Cirrhosis. Hepatology.

[5] Ramamonjisoa N., Ackerstaff E. (2017). Characterization of the Tumor Microenvironment and Tumor-Stroma Interaction by Non-Invasive Preclinical Imaging. Front. Oncol..

[6] Garin-Chesa P., Old L.J., Rettig W.J. (1990). Cell Surface Glycoprotein of Reactive Stromal Fibroblasts as a Potential Antibody Target in Human Epithelial Cancers. Proc. Natl. Acad. Sci. USA.

[7] Puré E. (2009). The Road to Integrative Cancer Therapies: Emergence of a Tumor-Associated Fibroblast Protease as a Potential Therapeutic Target in Cancer. Expert Opin. Ther. Targets.

[8] Hamson E.J., Keane F.M., Tholen S., Schilling O., Gorrell M.D. (2014). Understanding Fibroblast Activation Protein (FAP): Substrates, Activities, Expression and Targeting for Cancer Therapy. Proteom. –Clin. Appl..

[9] Goldstein L.A., Ghersi G., Piñeiro-Sánchez M.L., Salamone M., Yeh Y., Flessate D., Chen W.-T. (1997). Molecular Cloning of Seprase: A Serine Integral Membrane Protease from Human Melanoma. Biochim. Biophys. Acta -Mol. Basis Dis..

[10] Aertgeerts K., Levin I., Shi L., Snell G.P., Jennings A., Prasad G.S., Zhang Y., Kraus M.L., Salakian S., Sridhar V. (2005). Structural and Kinetic Analysis of the Substrate Specificity of Human Fibroblast Activation Protein α. J. Biol. Chem..

[11] Yu D.M.T., Yao T.-W., Chowdhury S., Nadvi N.A., Osborne B., Church W.B., McCaughan G.W., Gorrell M.D. (2010). The Dipeptidyl Peptidase IV Family in Cancer and Cell Biology. FEBS J..

[12] Verhulst E., Garnier D., De Meester I., Bauvois B. (2022). Validating Cell Surface Proteases as Drug Targets for Cancer Therapy: What Do We Know, and Where Do We Go?. Cancers.

[13] Keane F.M., Nadvi N.A., Yao T.W., Gorrell M.D. (2011). Neuropeptide Y, B-Type Natriuretic Peptide, Substance P and Peptide YY Are Novel Substrates of Fibroblast Activation Protein-α. FEBS J..

[14] Park J.E., Lenter M.C., Zimmermann R.N., Garin-Chesa P., Old L.J., Rettig W.J. (1999). Fibroblast Activation Protein, a Dual Specificity Serine Protease Expressed in Reactive Human Tumor Stromal Fibroblasts. J. Biol. Chem..

[15] Lee K.N., Jackson K.W., Christiansen V.J., Chung K.H., McKee P.A. (2004). A Novel Plasma Proteinase Potentiates Alpha2-Antiplasmin Inhibition of Fibrin Digestion. Blood.

[16] Lee K.N., Jackson K.W., Christiansen V.J., Dolence E.K., Mckee P.A. (2011). Enhancement of Fibrinolysis by Inhibiting Enzymatic Cleavage of Precursor A2-Antiplasmin. J. Thromb. Haemost..

[17] Aimes R.T., Zijlstra A., Hooper J.D., Ogbourne S.M., Sit M.-L., Fuchs S., Gotley D.C., Quigley J.P., Antalis T.M. (2003). Endothelial Cell Serine Proteases Expressed during Vascular Morphogenesis and Angiogenesis. Thromb. Haemost..

[18] Cao F., Wang S., Wang H., Tang W. (2018). Fibroblast Activation Protein-α in Tumor Cells Promotes Colorectal Cancer Angiogenesis via the Akt and ERK Signaling Pathways. Mol. Med. Rep..

[19] Jia J., Martin T.A., Ye L., Meng L., Xia N., Jiang W.G., Zhang X. (2018). Fibroblast Activation Protein-α Promotes the Growth and Migration of Lung Cancer Cells via the PI3K and Sonic Hedgehog Pathways. Int. J. Mol. Med..

[20] Huang Y., Simms A.E., Mazur A., Wang S., León N.R., Jones B., Aziz N., Kelly T. (2011). Fibroblast Activation Protein-α Promotes Tumor Growth and Invasion of Breast Cancer Cells through Non-Enzymatic Functions. Clin. Exp. Metastasis.

[21] Wen Z., Liu Q., Wu J., Xu B., Wang J., Liang L., Guo Y., Peng M., Zhao Y., Liao Q. (2019). Fibroblast Activation Protein α-Positive Pancreatic Stellate Cells Promote the Migration and Invasion of Pancreatic Cancer by CXCL1-Mediated Akt Phosphorylation. Ann. Transl. Med..

[22] Liu J., Huang C., Peng C., Xu F., Li Y., Yutaka Y., Xiong B., Yang X. (2018). Stromal Fibroblast Activation Protein Alpha Promotes Gastric Cancer Progression via Epithelial-Mesenchymal Transition through Wnt/ β-Catenin Pathway. BMC Cancer.

[23] Calais J. (2020). FAP: The Next Billion Dollar Nuclear Theranostics Target?. J. Nucl. Med..

[24] Jansen K., Heirbaut L., Verkerk R., Cheng J.D., Joossens J., Cos P., Maes L., Lambeir A.M., De Meester I., Augustyns K. (2014). Extended Structure-Activity Relationship and Pharmacokinetic Investigation of (4-Quinolinoyl)Glycyl-2-Cyanopyrrolidine Inhibitors of Fibroblast Activation Protein (FAP). J. Med. Chem..

[25] Lindner T., Loktev A., Altmann A., Giesel F., Kratochwil C., Debus J., Jäger D., Mier W., Haberkorn U. (2018). Development of Quinoline-Based Theranostic Ligands for the Targeting of Fibroblast Activation Protein. J. Nucl. Med..

[26] Loktev A., Lindner T., Burger E.M., Altmann A., Giesel F., Kratochwil C., Debus J., Marmé F., Jäger D., Mier W. (2019). Development of Fibroblast Activation Protein-Targeted Radiotracers with Improved Tumor Retention. J. Nucl. Med..

[27] Lindner T., Altmann A., Krämer S., Kleist C., Loktev A., Kratochwil C., Giesel F., Mier W., Marme F., Debus J. (2020). Design and Development of ^99m^Tc-Labeled FAPI Tracers for SPECT Imaging and ^188^Re Therapy. J. Nucl. Med..

[28] Roy J., Hettiarachchi S.U., Kaake M., Mukkamala R., Low P.S. (2020). Design and Validation of Fibroblast Activation Protein Alpha Targeted Imaging and Therapeutic Agents. Theranostics.

[29] Moon E.S., Elvas F., Vliegen G., De Lombaerde S., Vangestel C., De Bruycker S., Bracke A., Eppard E., Greifenstein L., Klasen B. (2020). Targeting Fibroblast Activation Protein (FAP): Next Generation PET Radiotracers Using Squaramide Coupled Bifunctional DOTA and DATA^5m^ Chelators. EJNMMI Radiopharm. Chem..

[30] Moon E.S., Van Rymenant Y., Battan S., De Loose J., Bracke A., Van der Veken P., De Messter I., Rosch F. (2021). In Vitro Evaluation of the Squaramide-Conjugated Fibroblast Activation Protein Inhibitor-Based Agents AAZTA^5^.SA.FAPi and DOTA.SA.FAPi. Molecules.

[31] Moon E.S., Ballal S., Yadav M.P., Bal C., Rymenant Y. (2021). Van Fibroblast Activation Protein (FAP) Targeting Homodimeric FAP Inhibitor Radiotheranostics: A Step to Improve Tumor Uptake and Retention Time. Am. J. Nucl. Med. Mol. Imaging.

[32] Toms J., Kogler J., Maschauer S., Daniel C., Schmidkonz C., Kuwert T., Prante O. (2020). Targeting Fibroblast Activation Protein: Radiosynthesis and Preclinical Evaluation of an ^18^F-Labeled FAP Inhibitor. J. Nucl. Med..

[33] Lindner T., Altmann A., Giesel F., Kratochwil C., Kleist C., Krämer S., Mier W., Cardinale J., Kauczor H.U., Jäger D. (2021). ^18^F-Labeled Tracers Targeting Fibroblast Activation Protein. EJNMMI Radiopharm. Chem..

[34] Slania S.L., Das D., Lisok A., Du Y., Jiang Z., Mease R.C., Rowe S.P., Nimmagadda S., Yang X., Pomper M.G. (2021). Imaging of Fibroblast Activation Protein in Cancer Xenografts Using Novel (4-Quinolinoyl)-Glycyl-2-Cyanopyrrolidine-Based Small Molecules. J. Med. Chem..

[35] Kratochwil C., Flechsig P., Lindner T., Abderrahim L., Altmann A., Mier W., Adeberg S., Rathke H., Röhrich M., Winter H. (2019). ^68^Ga-FAPI PET/CT: Tracer Uptake in 28 Different Kinds of Cancer. J. Nucl. Med..

[36] Giesel F.L., Kratochwil C., Lindner T., Marschalek M.M., Loktev A., Lehnert W., Debus J., Jäger D., Flechsig P., Altmann A. (2019). ^68^Ga-FAPI PET/CT: Biodistribution and Preliminary Dosimetry Estimate of 2 DOTA-Containing FAP-Targeting Agents in Patients with Various Cancers. J. Nucl. Med..

[37] Giesel F.L., Kratochwil C., Schlittenhardt J., Dendl K., Eiber M., Staudinger F., Kessler L., Fendler W.P., Lindner T., Koerber S.A. (2021). Head-to-Head Intra-Individual Comparison of Biodistribution and Tumor Uptake of ^68^Ga-FAPI and ^18^F-FDG PET/CT in Cancer Patients. Eur. J. Nucl. Med. Mol. Imaging.

[38] Wegen S., van Heek L., Linde P., Claus K., Akuamoa-Boateng D., Baues C., Sharma S.J., Schomäcker K., Fischer T., Roth K.S. (2022). Head-to-Head Comparison of [^68^Ga]Ga-FAPI-46-PET/CT and [^18^F]F-FDG-PET/CT for Radiotherapy Planning in Head and Neck Cancer. Mol. Imaging Biol..

[39] Ballal S., Yadav M.P., Moon E.S., Kramer V.S., Roesch F., Kumari S., Bal C. (2021). First-In-Human Results on the Biodistribution, Pharmacokinetics, and Dosimetry of [^177^Lu]Lu-DOTA.SA.FAPi and [^177^Lu]Lu-DOTAGA.(SA.FAPi)2. Pharmaceuticals.

[40] Ballal S., Yadav M.P., Kramer V., Moon E.S., Roesch F., Tripathi M., Mallick S., ArunRaj S.T., Bal C. (2021). A Theranostic Approach of [^68^Ga]Ga-DOTA.SA.FAPi PET/CT-Guided [^177^Lu]Lu-DOTA.SA.FAPi Radionuclide Therapy in an End-Stage Breast Cancer Patient: New Frontier in Targeted Radionuclide Therapy. Eur. J. Nucl. Med. Mol. Imaging.

[41] Zboralski D., Hoehne A., Bredenbeck A., Schumann A., Nguyen M., Schneider E., Ungewiss J., Paschke M., Haase C., von Hacht J.L. (2022). Preclinical Evaluation of FAP-2286 for Fibroblast Activation Protein Targeted Radionuclide Imaging and Therapy. Eur. J. Nucl. Med. Mol. Imaging.

[42] Baum R.P., Schuchardt C., Singh A., Chantadisai M., Robiller F.C., Zhang J., Mueller D., Eismant A., Almaguel F., Zboralski D. (2022). Feasibility, Biodistribution, and Preliminary Dosimetry in Peptide-Targeted Radionuclide Therapy of Diverse Adenocarcinomas Using ^177^Lu-FAP-2286: First-in-Humans Results. J. Nucl. Med..

[43] Notni J., Hermann P., Havlíčková J., Kotek J., Kubíček V., Plutnar J., Loktionova N., Riss P.J., Rösch F., Lukeš I. (2010). A Triazacyclononane-Based Bifunctional Phosphinate Ligand for the Preparation of Multimeric ^68^Ga Tracers for Positron Emission Tomography. Chem.—A Eur. J..

[44] Notni J., Pohle K., Wester H.J. (2013). Be Spoilt for Choice with Radiolabelled RGD Peptides: Preclinical Evaluation of ^68^Ga-TRAP(RGD)3. Nucl. Med. Biol..

[45] Šimeček J., Hermann P., Havlíčková J., Herdtweck E., Kapp T.G., Engelbogen N., Kessler H., Wester H.-J., Notni J. (2013). A Cyclen-Based Tetraphosphinate Chelator for the Preparation of Radiolabeled Tetrameric Bioconjugates. Chemistry.

[46] Wurzer A., Vágner A., Horváth D., Fellegi F., Wester H.J., Kálmán F.K., Notni J. (2018). Synthesis of Symmetrical Tetrameric Conjugates of the Radiolanthanide Chelator DOTPI for Application in Endoradiotherapy by Means of Click Chemistry. Front. Chem..

[47] Zia N.A., Cullinane C., Van Zuylekom J.K., Waldeck K., McInnes L.E., Buncic G., Haskali M.B., Roselt P.D., Hicks R.J., Donnelly P.S. (2019). A Bivalent Inhibitor of Prostate Specific Membrane Antigen Radiolabeled with Copper-64 with High Tumor Uptake and Retention. Angew. Chem. Int. Ed..

[48] Zhao L., Niu B., Fang J., Pang Y., Li S., Xie C., Sun L., Zhang X., Guo Z., Lin Q. (2022). Synthesis, Preclinical Evaluation, and a Pilot Clinical PET Imaging Study of ^68^Ga-Labeled FAPI Dimer. J. Nucl. Med..

[49] Younis M.H., Lan X., Cai W. (2022). PET with a ^68^Ga-Labeled FAPI Dimer: Moving towards Theranostics. J. Nucl. Med..

[50] Galbiati A., Zana A., Bocci M., Millul J., Elsayed A., Mock J., Neri D., Cazzamalli S. (2022). A Novel Dimeric FAP-Targeting Small Molecule-Radio Conjugate with High and Prolonged Tumour Uptake. J. Nucl. Med..

[51] Li H., Ye S., Li L., Zhong J., Yan Q., Zhong Y., Feng P., Hu K. (2022). ^18^F- or ^177^Lu-Labeled Bivalent Ligand of Fibroblast Activation Protein with High Tumor Uptake and Retention. Eur. J. Nucl. Med. Mol. Imaging.

[52] Ballal S., Yadav M.P., Moon E.S., Roesch F., Kumari S., Agarwal S., Tripathi M., Sahoo R.K., Mangu B.S., Tupalli A. (2022). Novel Fibroblast Activation Protein Inhibitor-Based Targeted Theranostics for Radioiodine-Refractory Differentiated Thyroid Cancer Patients: A Pilot Study. Thyroid.

[53] Eppard E., Wuttke M., Nicodemus P.L., Rösch F. (2014). Ethanol-Based Post-Processing of Generator-Derived ^68^Ga towards Kit-Type Preparation of ^68^Ga-Radiopharmaceuticals. J. Nucl. Med..

[54] De Decker A., Vliegen G., Van Rompaey D., Peeraer A., Bracke A., Verckist L., Jansen K., Geiss-Friedlander R., Augustyns K., De Winter H. (2019). Novel Small Molecule-Derived, Highly Selective Substrates for Fibroblast Activation Protein (FAP). ACS Med. Chem. Lett..

[55] Van Rymenant Y., Tanc M., Van Elzen R., Bracke A., De Wever O., Augustyns K., Lambeir A.M., Kockx M., De Meester I., Van Der Veken P. (2021). In Vitro and In Situ Activity-Based Labeling of Fibroblast Activation Protein with UAMC1110-Derived Probes. Front. Chem..

[56] De Meester I., Vanhoof G., Lambeir A.-M., Scharpé S. (1996). Use of Immobilized Adenosine Deaminase (EC 3.5.4.4) for the Rapid Purification of Native Human CD26/Dipeptidyl Peptidase IV (EC 3.4.14.5). J. Immunol. Methods.

[57] Benramdane S., De Loose J., Beyens O., Van Rymenant Y., Vliegen G., Augustyns K., De Winter H., De Meester I., der Veken P. (2022). Vildagliptin-Derived Dipeptidyl Peptidase 9 (DPP9) Inhibitors: Identification of a DPP8/9-Specific Lead. ChemMedChem.

[58] Ballal S., Yadav M.P., Moon E.S., Kramer V.S., Roesch F., Kumari S., Tripathi M., ArunRaj S.T., Sarswat S., Bal C. (2021). Biodistribution, Pharmacokinetics, Dosimetry of [^68^Ga]Ga-DOTA.SA.FAPi, and the Head-to-Head Comparison with [^18^F]F-FDG PET/CT in Patients with Various Cancers. Eur. J. Nucl. Med. Mol. Imaging.

[59] Kelly J., Amor-Coarasa A., Sweeney E., Wilson J., Causey P., Babich J. (2021). A Consensus Time for Performing Quality Control of 225Ac-Labeled Radiopharmaceuticals. https://assets.researchsquare.com/files/rs-39342/v2_covered.pdf?c=1631863217.

[60] Aime S., Barge A., Botta M., Fasano M., Ayala J.D., Bombieri G. (1996). Crystal Structure and Solution Dynamics of the Lutetium(III) Chelate of DOTA. Inorg. Chim. Acta.

[61] Khreish F., Ebert N., Ries M., Maus S., Rosar F., Bohnenberger H., Stemler T., Saar M., Bartholomä M., Ezziddin S. (2020). ^225^Ac-PSMA-617/^177^Lu-PSMA-617 Tandem Therapy of Metastatic Castration-Resistant Prostate Cancer: Pilot Experience. Eur. J. Nucl. Med. Mol. Imaging.

[62] Ilhan H., Gosewisch A., Böning G., Völter F., Zacherl M., Unterrainer M., Bartenstein P., Todica A., Gildehaus F.J. (2021). Response to ^225^Ac-PSMA-I&T after Failure of Long-Term ^177^Lu-PSMA RLT in MCRPC. Eur. J. Nucl. Med. Mol. Imaging.

[63] Ballal S., Yadav M.P., Bal C., Sahoo R.K., Tripathi M. (2020). Broadening Horizons with ^225^Ac-DOTATATE Targeted Alpha Therapy for Gastroenteropancreatic Neuroendocrine Tumour Patients Stable or Refractory to ^177^Lu-DOTATATE PRRT: First Clinical Experience on the Efficacy and Safety. Eur. J. Nucl. Med. Mol. Imaging.

[64] Watabe T., Liu Y., Kaneda-Nakashima K., Shirakami Y., Lindner T., Ooe K., Toyoshima A., Nagata K., Shimosegawa E., Haberkorn U. (2020). Theranostics Targeting Fibroblast Activation Protein in the Tumor Stroma: ^64^Cu- And ^225^Ac-Labeled FAPI-04 in Pancreatic Cancer Xenograft Mouse Models. J. Nucl. Med..

[65] Thiele N.A., Wilson J.J. (2018). Actinium-225 for Targeted α Therapy: Coordination Chemistry and Current Chelation Approaches. Cancer Biother. Radiopharm..

[66] Robertson A.K.H., Ramogida C.F., Schaffer P., Radchenko V. (2018). Development of ^225^Ac Radiopharmaceuticals: TRIUMF Perspectives and Experiences. Curr. Radiopharm..

[67] Ansems M., Span P.N. (2020). The Tumor Microenvironment and Radiotherapy Response; a Central Role for Cancer-Associated Fibroblasts. Clin. Transl. Radiat. Oncol..

[68] Domogauer J.D., de Toledo S.M., Howell R.W., Azzam E.I. (2021). Acquired Radioresistance in Cancer Associated Fibroblasts Is Concomitant with Enhanced Antioxidant Potential and DNA Repair Capacity. Cell Commun. Signal..

[69] Wang Z., Tang Y., Tan Y., Wei Q., Yu W. (2019). Cancer-Associated Fibroblasts in Radiotherapy: Challenges and New Opportunities. Cell Commun. Signal..

[70] Cirri P., Chiarugi P. (2011). Cancer Associated Fibroblasts: The Dark Side of the Coin. Am. J. Cancer Res..

[71] De Veirman K., Rao L., De Bruyne E., Menu E., Van Valckenborgh E., Van Riet I., Frassanito M.A., Di Marzo L., Vacca A., Vanderkerken K. (2014). Cancer Associated Fibroblasts and Tumor Growth: Focus on Multiple Myeloma. Cancers.

