# Supplementary material for: Novel Generation of FAP Inhibitor-Based Homodimers for Improved Application in Radiotheranostics
Source: Cancers (Basel). 2023 Mar 21;15(6):1889. doi: 10.3390/cancers15061889 (PMC10047490; doi:10.3390/cancers15061889)
Supplement: Supplementary file 1 [file cancers-15-01889-s001.zip › cancers-2169568-supplementary.pdf]

cancers

MDPI

Article

Novel Generation of FAP Inhibitor-Based Homodimers for Improved Application in Radiotheranostics

Marcel Martin [1,*], Sanjana Ballal [2], Madhav Prasad Yadav [2], Chandrasekhar Bal [2], Yentl Van Rymenant [3], Joni De Loose [3], Emile Verhulst [3], Ingrid De Meester [3], Pieter Van Der Veken [4] and Frank Roesch [1]

[1] Department of Chemistry—TRIGA Site, Johannes Gutenberg University Mainz, 55128 Mainz, Germany; froesch@uni-mainz.de
[2] Department of Nuclear Medicine, All India Institute of Medical Sciences, New Delhi 110029, India; mail.sanjanaballal87@gmail.com (S.B.); madhav_yadav2000@yahoo.com (M.P.Y.); csbal@hotmail.com (C.B.)
[3] Laboratory of Medical Biochemistry, Department of Pharmaceutical Sciences, University of Antwerp, 2610 Wilrijk, Belgium; yentl.vanrymenant@uantwerpen.be (Y.V.R.); joni.deloose@uantwerpen.be (J.D.L.); emile.verhulst@uantwerpen.be (E.V.) ingrid.demeester@uantwerpen.be (I.D.M.)
[4] Laboratory of Medicinal Chemistry, Department of Pharmaceutical Sciences, University of Antwerp, 2610 Wilrijk, Belgium; pieter.vanderveken@uantwerpen.be
* Correspondence: mamarcel@uni-mainz.de; Tel.: +49-6131-3921975

Supporting Information

Organic synthesis of FAPi-NH$_2$

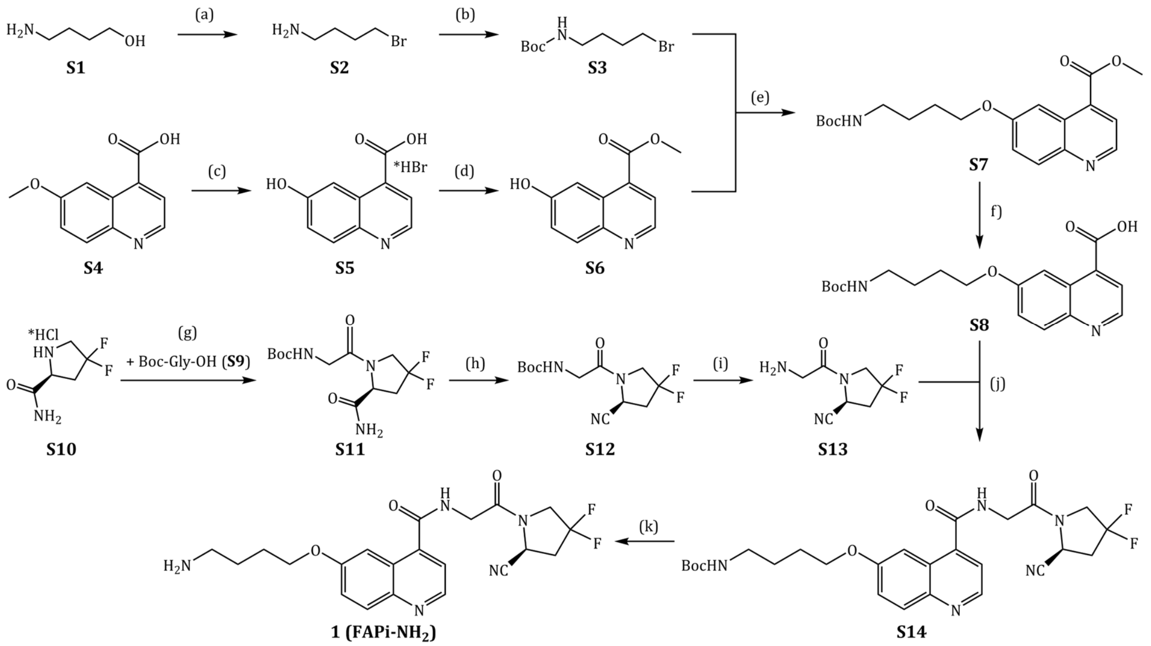

Figure S1. Organic synthesis of FAPi-NH$_2$ **1**: (a) HBr (47%), 126 °C, 4 h, 96%; (b) Boc$_2$O, TEA, THF, RT, 19 h, 66%; (c) HBr (47%), 126 °C, 1 d, 100%; (d) SOCl$_2$, MeOH, 0 °C–RT, 2 d, 100%; (e) Cs$_2$CO$_3$, DMF, 70 °C, 1 d, 59%; (f) 1 M LiOH, 1,4-dioxane, RT, 4 h, 57%; (g) HATU, DIPEA, DCM/DMF (1:1), RT, 19 h, 86%; (h) TFAA, pyridine, THF, DCM, RT, 3 h, 81%; (i) TFA, MeCN, RT, 5 h, 100%; (j) HBTU, HOBt, DIPEA, DMF, RT, 1 d, 72%; (k) 4 M HCl in 1,4-dioxane, MeCN, 0 °C–RT, 7 h, 100%.

4-Bromobutylamine (**S2**)

47% hydrobromic acid (HBr, 70 mL) was slowly added to 4-aminobutanol (**S1**, 5.39 g, 60.5 mmol, 1.00 eq) and then heated for 4 hours under reflux. Then, the reaction mixture was concentrated *in vacuo*. **S2** was obtained as a colorless solid (13.5 g, 58.0 mmol, 96%)

and used directly in the next step without any further purification. ^{1}H-NMR (400 MHz, MeOD): δ [ppm] = 3.51 (t, *J* = 6.4 Hz, 2H), 2.98 (t, *J* = 7.6 Hz, 2H), 2.02 – 1.73 (m, 4H). MS (ESI$^+$): m/z (%) = 152.0 (100, [M+H]$^+$), 154.0 (98, [M+H]$^+$), calculated for $C_4H_{10}BrN$: 151.00 [M].

tert-Butyl (4-bromobutyl)carbamate (**S3**)

4-Bromobutylamine (**S2**, 7.01 g, 30.1 mmol, 1.00 eq) and di-*tert*-butyl dicarbonate (Boc_2O, 7.34 g, 33.6 mmol, 1.12 eq) were dissolved in dry tetrahydrofuran (THF, 34 mL) under argon atmosphere. Triethylamine (TEA, 4.6 mL, 36.1 mmol, 1.20 eq) was then added, followed by MeOH (36 mL) to turn the suspension into a clear solution again. The solution was stirred overnight at RT. The solvent was removed *in vacuo* and diluted HBr was added to the residue until pH = 2.5. The aqueous solution was extracted with diethyl ether (Et_2O, 5×80 mL) and the combined organic phases were washed once with $NaHCO_3$ (10 mL) and Brine (10 mL) and then dried over Na_2SO_4. The solvent was removed under reduced pressure and after column chromatography (cyclohexane/ethyl acetate (CH/EA, 5:1)) **S3** was obtained as a colorless solid (5.08 g, 20.2 mmol, 66%). ^{1}H-NMR (400 MHz, $CDCl_3$): δ [ppm] = 3.36 − 3.21 (m, 4H), 1.86 - 1.76 (m, 4H), 1.43 (s, 9H). MS (ESI$^+$): m/z (%) = 196.0 (100, [M−^{t}Bu+H]$^+$), 198.0 (100, [M−^{t}Bu+H]$^+$), calculated for $C_9H_{18}BrNO_2$: 251.05 [M].

6-Hydroxyquinoline-4-carboxylic acid hydrobromide (**S5**)

6-Methoxyquinoline-4-carboxylic acid (**S4**, 2.46 g, 12.1 mmol, 1.00 eq) was dissolved in 47% HBr (28.2 mL, 242.4 mmol, 20 eq) and heated for one day under reflux. After cooling to RT, the hydrobromic acid was partially removed *in vacuo* and the precipitate was then filtered off and washed first with cold EA (20 mL) and then with cold EA/MeOH (9:1, 10 mL). **S5** was obtained as a yellow solid (3.25 g, 12.1 mmol, 100%). ^{1}H-NMR (400 MHz, MeOD): δ [ppm] = 9.05 (d, *J* = 5.6 Hz, 1H), 8.41 (d, *J* = 5.6 Hz, 1H), 8.33 (d, *J* = 2.6 Hz, 1H), 8.19 (d, *J* = 9.3 Hz, 1H), 7.77 (dd, *J* = 9.3, 2.6 Hz, 1H). MS (ESI$^+$): m/z (%) = 190.0 (100, [M+H]$^+$), 191.0 (12, [M+H]$^+$), calculated for $C_{10}H_8BrNO_3$: 189.04 [M].

6-Hydroxyquinoline-4-carboxylic acid methyl ester (**S6**)

Dry MeOH (20 mL) was cooled to 0°C under argon atmosphere and $SOCl_2$ (4.43 mL, 61.1 mmol, 5.05 eq) was added dropwise. 6-Hydroxyquinoline-4-carboxylic acid hydrobromide (**S5**, 3.25 g, 12.1 mmol, 1.00 eq) was dissolved in dry MeOH (20 mL) and also cooled to 0°C under argon atmosphere. Then, the $SOCl_2$-MeOH solution was added dropwise to **S5**. At first, the reaction solution was allowed to warm to RT and then heated under reflux for one day. $SOCl_2$ (2.91 g, 24.4 mmol, 2.02 eq) and MeOH (20 mL) were again combined at 0 °C and added to the reaction mixture at RT. The solution was heated at reflux for an additional day. The step was repeated one more time and after another 4 hours of heating under reflux, the solvent was removed under reduced pressure. **S6** was obtained as a yellow solid (2.46 g, 12.1 mmol, 100%) which was used in the next step without any further purification. ^{1}H-NMR (400 MHz, MeOD): δ [ppm] = 9.02 (d, *J* = 5.5 Hz, 1H), 8.38 (d, *J* = 5.5 Hz, 1H), 8.24 (d, *J* = 2.6 Hz, 1H), 8.17 (d, *J* = 9.3 Hz, 1H), 7.75 (dd, *J* = 9.3, 2.6 Hz, 1H), 4.09 (s, 3H). MS (ESI$^+$): m/z (%) = 204.0 (100, [M+H]$^+$), 205.1 (12, [M+H]$^+$), calculated for $C_{11}H_9NO_3$: 203.06 [M].

Methyl 6-(4-((*tert*-butoxycarbonyl)amino)butoxy)quinoline-4-carboxylate (**S7**, Boc-Chino-COOMe)

6-Hydroxyquinoline-4-carboxylic acid methyl ester (**S6**, 2.46 g, 12.1 mmol, 1.00 eq) and Cs_2CO_3 (4.37 g, 13.4 mmol, 1.25 eq) were suspended in dry DMF (55 mL). The reaction solution was heated to 70°C. Then, *tert*-butyl (4-bromobutyl)carbamate (**S3**, 3.76 g, 14.9 mmol, 1.22 eq), dissolved in dry DMF (80 mL), was dropped into the reaction mixture.

The solution was stirred for 3 hours at 70°C before some more **S3** (1.23 g, 4.88 mmol, 0.4 eq., again dissolved in dry DMF (20 mL), was added. It was stirred overnight at 70°C. After another addition of **S3** (308 mg, 1.22 mmol, 0.1 eq) and another 3 hours at 70°C, the solvent was removed *in vacuo* and the residue was taken up in diluted HBr (150 mL, pH = 2.6). It was extracted with EA (5×80 mL), and the organic phase was washed with Brine (20 mL) and dried over Na_2SO_4. The solvent was removed under reduced pressure and the product **S7** was purified via column chromatography ($CHCl_3$/MeOH, 100:1) to give a yellowish solid (2.68 g, 7.17 mmol, 59%). ^{1}H-NMR (400 MHz, $CDCl_3$): δ [ppm] = 8.84 (d, *J* = 4.5 Hz, 1H), 8.21 (d, *J* = 2.8 Hz, 1H), 8.07 (d, *J* = 9.2 Hz, 1H), 7.92 (d, *J* = 4.5 Hz, 1H), 7.41 (dd, *J* = 9.2, 2.8 Hz, 1H), 4.66 (s, 1H), 4.15 (t, *J* = 6.2 Hz, 2H), 4.02 (s, 3H), 3.23 (q, *J* = 6.7 Hz, 2H), 1.90 (tt, *J* = 8.6, 6.0 Hz, 2H), 1.77 – 1.68 (m, 2H), 1.44 (s, 9H). MS (ESI$^+$): m/z (%) = 375.2 (100, $[M+H]^+$), 376.2 (23, $[M+H]^+$), calculated for $C_{20}H_{26}N_2O_5$: 374.18 [M].

6-(4-((*tert*-Butoxycarbonyl)amino)butoxy)quinoline-4-carboxylic acid (**S8**, Boc-Chino-COOH)

Boc-Chino-COOMe (**S7**, 3.34 g, 8.92 mmol, 1.00 eq) was dissolved in 1,4-dioxane (40 mL) and 1 M LiOH (17.8 mL, 17.8 mmol, 2.00 eq) was added and stirred for 4 hours at RT. The organic solvent was removed *in vacuo* and the solution was adjusted to pH = 3.5 with 1 M HCl. The aqueous solution was extracted with EA (8×80 mL), the organic phase was dried over Na_2SO_4 and the solvent was removed under reduced pressure. **S8** was obtained as a yellowish solid (1.82 g, 5.05 mmol, 57%). ^{1}H-NMR (400 MHz, DMSO-d_6): δ [ppm] = 8.86 (d, *J* = 4.5 Hz, 1H), 8.15 (d, *J* = 2.8 Hz, 1H), 8.02 (d, *J* = 9.3 Hz, 1H), 7.92 (d, *J* = 4.4 Hz, 1H), 7.49 (dd, *J* = 9.2 Hz, 2.8 Hz, 1H), 6.87 (t, *J* = 5.8 Hz, 1H), 4.10 (t, *J* = 6.3 Hz, 2H), 3.00 (q, *J* = 6.6 Hz, 2H), 1.78 (q, *J* = 11.8, 6.5 Hz, 2H), 1.62 – 1.51 (m, 2H), 1.37 (s, 9H). MS (ESI$^+$): m/z (%) = 261.1 (20, $[M{-}Boc+H]^+$), 361.2 (100, $[M+H]^+$), 362.2 (22, $[M+H]^+$), calculated for $C_{19}H_{24}N_2O_5$: 360.17 [M].

tert-Butyl (S)-(2-(2-carbamoyl-4,4-difluoropyrrolidin-1-yl)-2-oxoethyl)carbamate (**S11**, Boc-Gly-Pro-$CONH_2$)

Boc-Gly-OH (**S9**, 1.38 g, 7.88 mmol, 1.05 eq) and HBTU (3.12 g, 8.20 mmol, 1.1 eq) were dissolved in dry dichloromethane (DCM, 8 mL) and DMF (8 mL) under argon atmosphere. DIPEA (1.53mL, 8.97 mmol, 1.20 eq) was added and the solution was stirred for one hour at RT. In another reaction vessel, 4,4-difluoro-L-prolinamide hydrochloride (**S10**, 1.40 g, 7.50 mmol, 1.00 eq) and DIPEA (2.54 mL, 14.90 mmol, 2.00 eq) were dissolved in dry DCM (5 mL) and DMF (5 mL). The solutions were combined and stirred overnight at RT. The precipitate was filtered off and the filtrate was cooled overnight to complete the precipitation. The two precipitates were combined and **S6** was obtained as a colorless solid (1.97 g, 6.41 mmol, 86%). ^{1}H-NMR (400 MHz, DMSO-d_6): δ [ppm] = 7.40 (s, 1H), 7.16 (s, 1H), 6.87 (dt, *J* = 10.4, 5.8 Hz, 1H), 4.45 (dd, *J* = 9.0 Hz, 1H), 4.15 – 3.85 (m, 2H), 3.86 - 3.63 (m, 2H), 2.81 – 2.27 (m, 2H), 1.37z (s, 9H). MS (ESI$^+$): m/z (%) = 207.8 (62, $[M{-}Boc+H]^+$), 251.8 (100, $[M{-}{}^tBu+H]^+$), 307.9 (39, $[M+H]^+$), 329.9 (24, $[M+Na]^+$), calculated for $C_{12}H_{19}F_2N_3O_4$: 307.13 [M].

tert-Butyl (S)-(2-(2-cyano-4,4-difluoropyrrolidin-1-yl)-2-oxoethyl)carbamate (**S12**, Boc-Gly-Pro-CN)

Boc-Gly-Pro-$CONH_2$ (**S11**, 1.97 g, 6.41 mmol, 1.00 eq) was dissolved in dry THF (50 mL) under argon atmosphere and cooled to 0°C. Pyridine (4.1 mL, 51.3 mmol, 8.00 eq) was added. In another reaction vessel, Trifluoracetic anhydride (TFAA, 2.7 mL, 19.2 mmol, 3.00 eq) was dissolved in dry DCM (35 mL) and slowly dropped to the reaction solution. The reaction solution was allowed to warm to RT while stirring for another 3 hours. Then, 1 M HCl (80 mL) was added and the aqueous solution was extracted with DCM (5x80 mL). The combined organic phases were washed once with Na_2CO_3 (10 mL)

and Brine (10 mL) and then dried over Na_2SO_4. The solvent was removed *in vacuo* and the product **S12** was purified via column chromatography (CH/EA 3:2) to give a colorless solid (1.49 g, 4.81 mmol, 81%). ^{1}H-NMR (400 MHz, $CDCl_3$): δ [ppm] = 5.35 (s, 1H), 4.97 (t, *J* = 6.5 Hz, 1H), 4.04 – 3.78 (m, 4H), 2.81 – 2.69 (m, 2H), 1.45 (s, 9H). MS (ESI$^+$): m/z (%) = 190.0 (31, [M−Boc+H]$^+$), 233.9 (100, [M−^{t}Bu+H]$^+$), calculated for $C_{12}H_{17}F_2N_2O_3$: 289.12 [M].

(S)-4,4-Difluoro-1-glycylpyrrolidine-2-carbonitrile trifluoroacetic acid (**S13**, Gly-Pro-CN)

Boc-Gly-Pro-CN (**S12**, 1.15 g, 3.97 mmol, 1.00 eq) was dissolved in dry MeCN (2 mL) and TFA (2 mL) was slowly added under argon atmosphere. After stirring for 5 hours at RT the solvent was removed under reduced pressure and codistilled with MeOH (5×25 mL). **S13** was obtained as a yellowish oil (1.20 g, 3.97 mmol, 100%) which was used in the next step without any further purification. ^{1}H-NMR (400 MHz, MeOD): δ [ppm] = 8.25 (s, 2H), 5.22 – 5.06 (m, 1H), 4.33 – 3.75 (m, 4H), 3.02 – 2.73 (m, 2H). MS (ESI$^+$): m/z (%) = 189.9 (100, [M+H]$^+$), 231.0 (20, [M+MeCN+H]$^+$), calculated for $C_7H_9F_2N_3O$: 189.07 [M].

tert-Butyl (S)-(4-((4-((2-(2-cyano-4,4-difluoropyrrolidin-1-yl)-2-oxoethyl)carbamoyl)quinolin-6-yl)oxy)butyl)carbamate (**S14**, FAPi-NHBoc)

Boc-Chino-COOH (**S8**, 1.64 g, 4.55 mmol, 1.15 eq) and DIPEA (0.93 mL, 5.46 mmol, 1.37 eq) were dissolved in dry DMF (16 mL) under argon atmosphere. HOBt (0.68 g, 5.01 mmol, 1.26 eq) and HBTU (1.90 g, 5.01 mmol, 1.26 eq) were then added and the reaction mixture was stirred for one hour at RT. Gly-Pro-CN (**S13**, 1.20 g, 3.97 mmol, 1.00 eq), also dissolved in dry DMF (10 mL) and mixed with DIPEA (1.93 mL, 11.38 mmol, 2.86 eq), was then added and the whole reaction mixture was stirred for one day at RT. Then the solvent was removed *in vacuo* and the residue was taken up in EA (100 mL). The organic phase was washed with 1 M citric acid, saturated Na_2CO_3 and Brine (10 mL each). The aqueous phase was extracted with EA (3×100 mL) and the combined organic extracts were dried over Na_2SO_4. The solvent was removed under reduced pressure and the product **S14** purified via column chromatography ($CHCl_3$/MeOH, 100:3) to give a colorless solid (1.74 g, 3.27 mmol, 72%). ^{1}H-NMR (400 MHz, MeOD): δ [ppm] = 8.74 (d, *J* = 4.4 Hz, 1H), 7.96 (d, *J* = 9.3 Hz, 1H), 7.93 – 7.88 (m, 1H), 7.56 (d, *J* = 4.4 Hz, 1H), 7.46 (dd, *J* = 9.3, 2.7 Hz, 1H), 5.15 (dd, *J* = 9.4, 3.1 Hz, 1H), 4.39 – 3.98 (m, 8H), 3.19 – 3.09 (m, 2H), 3.02 - 2.70 (m, 2H), 1.94 – 1.83 (m, 2H), 1.76 – 1.65 (m, 2H), 1.43 (s, 9H). MS (ESI$^+$): m/z (%) = 432.0 (33, [M−Boc+H]$^+$), 476.1 (46, [M−^{t}Bu+H]$^+$), 532.4 (100, [M+H]$^+$), calculated for $C_{26}H_{31}F_2N_5O_5$: 531.23 [M].

(S)-6-(4-Aminobutoxy)-N-(2-(2-cyano-4,4-difluoropyrrolidin-1-yl)-2-oxoethyl)quinoline-4-carboxamide (**1**, FAPi-NH_2)

FAPi-NHBoc (**S14**, 531.6 mg, 1.0 mmol, 1.0 eq) was dissolved in dry MeCN (10 mL) at 0 °C under argon atmosphere. 4 M HCl in 1,4-dioxane (5.0 mL, 5.0 mmol, 5.00 eq) was added and the reaction solution was slowly allowed to warm to RT. After 3 hours more 4 M HCl in 1,4-dioxane (2.5 mL, 2.5 mmol, 2.5 eq) was added. After another 4 hours it was diluted with MeCN (30 mL) and then completely concentrated *in vacuo*. FAPi-NH_2 **1** was obtained as a colorless solid (467 mg, 1.0 mmol, 100%). ^{1}H-NMR (400 MHz, MeOD): δ [ppm] = 9.10 (d, *J* = 5.5 Hz, 1H), 8.32 (d, *J* = 2.7 Hz, 1H), 8.24 (d, *J* = 9.3 Hz, 1H), 8.08 (d, *J* = 5.5 Hz, 1H), 7.86 (dd, *J* = 9.4, 2.6 Hz, 1H), 5.15 (dd, *J* = 9.4, 3.1 Hz, 1H), 4.48 – 4.33 (m, 4H), 4.32 – 4.07 (m, 2H), 3.06 (t, *J* = 6.5 Hz, 2H), 3.02 – 2.74 (m, 2H), 2.09 – 1.87 (m, 4H). MS (ESI$^+$): m/z (%) = 216.7 (100, [M+H]$^{2+}$), 237.2 (27, [M+MeCN+H]$^{2+}$), 432.1 (22, [M+H]$^+$), calculated for $C_{21}H_{23}O_5F_2N_5O_3$: 431.18 [M].

Analytical RP-HPLC

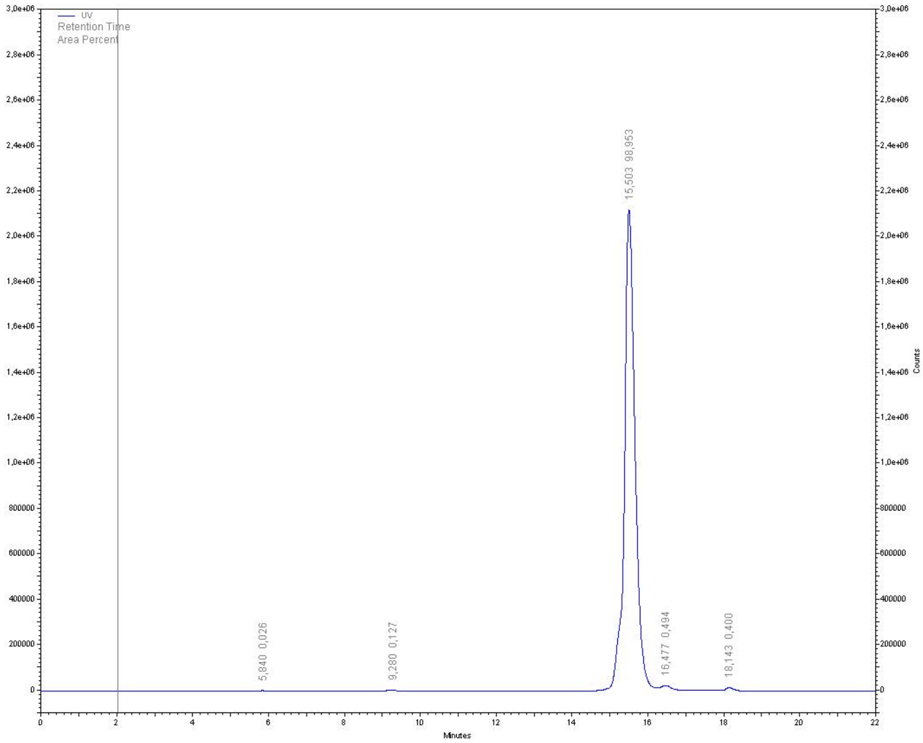

Figure S1. Analytical RP-HPLC of DOTAGA.Glu.(FAPi)$_2$ **7** (t_R = 15.5 min), 20-30% MeCN + 0.1% TFA (linear gradient) in 20 min, 5 mL/min: purity = 99.0%.

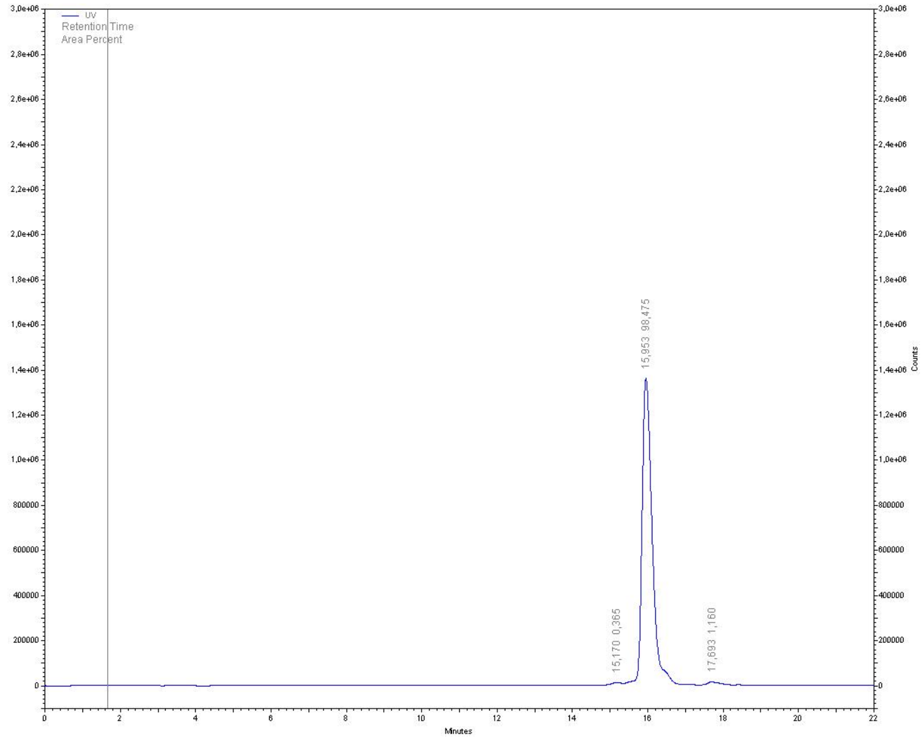

Figure S2. Analytical RP-HPLC of DO3A.Glu.(FAPi)$_2$ **11** (t_R = 16.0 min), 20-30% MeCN + 0.1% TFA (linear gradient) in 20 min, 5 mL/min: purity = 98.5%.

Radiosynthesis

The following Figures show additional labeling studies (reaction kinetics) as well as complex stability studies.

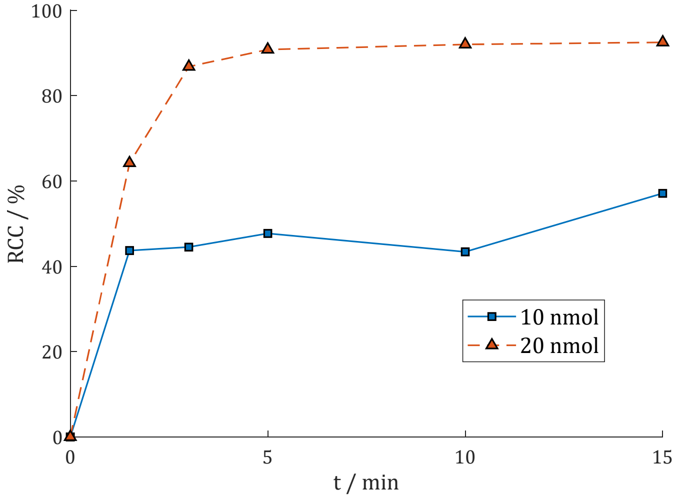

Figure S3. Reaction kinetics (radiochemical conversion (RCC) in %) of [^{68}Ga]Ga-DOTAGA.Glu.(FAPi)$_2$ **^{68}Ga-7** in 1 M AmOAc (pH = 4.5) at 95 °C with 100 MBq ^{68}Ga (n = 1).

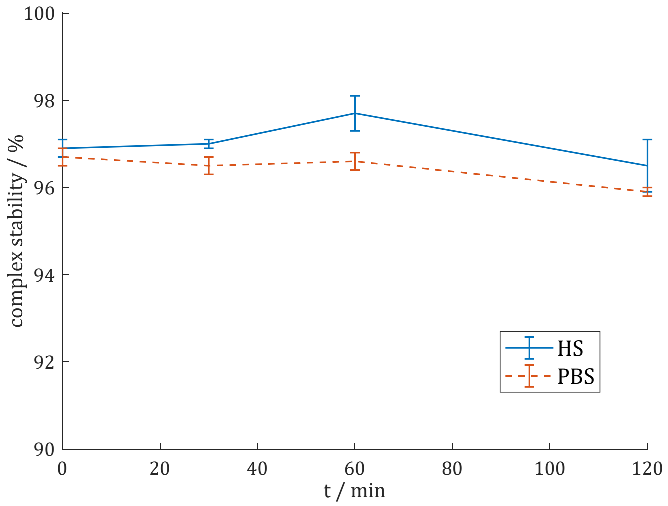

Figure S4. Complex stability of [^{68}Ga]Ga-DOTAGA.Glu.(FAPi)$_2$ **^{68}Ga-7** in human serum (HS) and phosphate-buffered saline (PBS) over 120 minutes (n = 3).

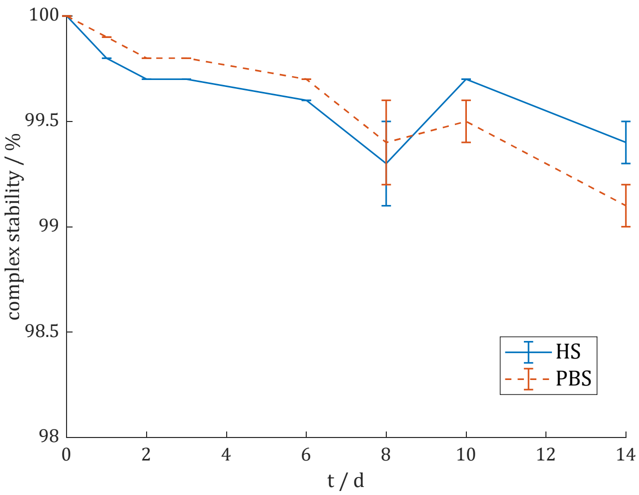

Figure S5. Complex stability of [^{177}Lu]Lu-DOTAGA.Glu.(FAPi)2 **[177]Lu-7** in human serum (HS) and phosphate-buffered saline (PBS) over 14 days (n = 3).

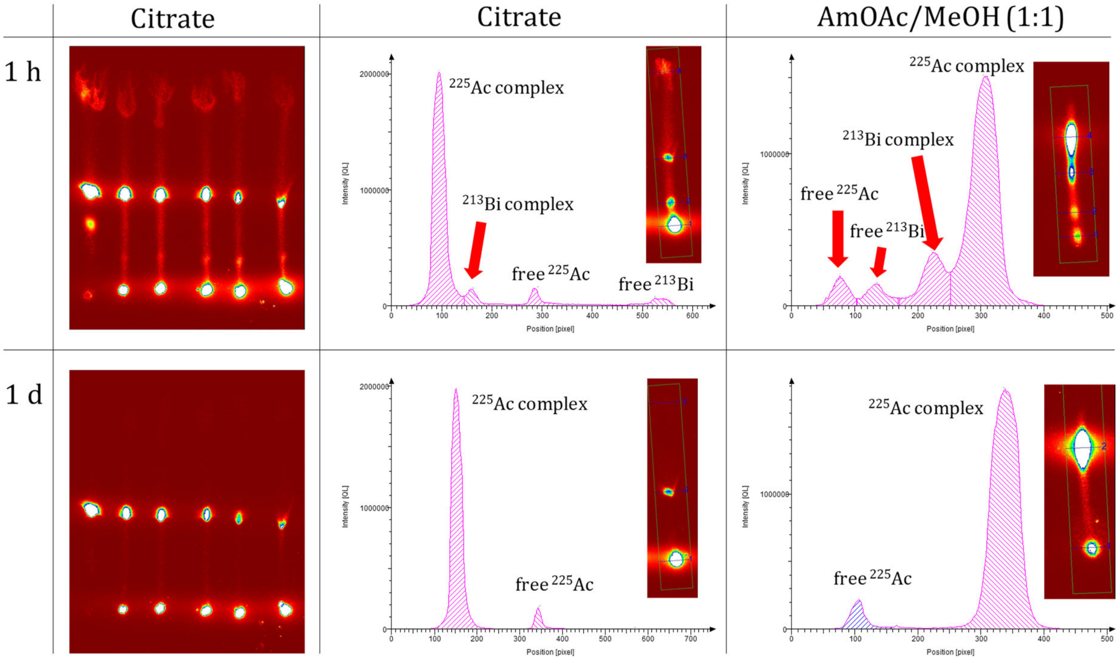

Figure S6. Exemplary reaction kinetic of [[225]Ac]Ac-DOTAGA.Glu.(FAPi)2 **[225]Ac-7** at 95 °C analyzed with 0.1 M citrate buffer (pH = 4.0) radio-TLC from 0–15 min (left to right). Exemplary profiles of citrate radio-TLC after 60 minutes at 95 °C are shown in the middle. The corresponding 1 M AmOAc (pH = 4.0)/MeOH (1:1) radio-TLCs are shown on the right. All TLCs were imaged at different time points after 1 hour (top) and 1 day (bottom). These measurements together make clear that: R_f([225]Ac-complex=product **[225]Ac-7**) = 0.0 and R_f(free [225]Ac) ≈ 0.5. The other two spots appearing in a) must be from one of the short-lived daughter nuclides, most likely [213]Bi since the spots are slowly decaying when measuring the TLC over several hours which fits the physical half-life and Bi is a trivalent metal that can be complexed by DOTA conjugates. Therefore, R_f(free [213]Bi) > 0.7 can be assigned to be free [213]Bi and R_f([213]Bi-complex) = 0.1–0.2 is considered to be the [213]Bi-complex.

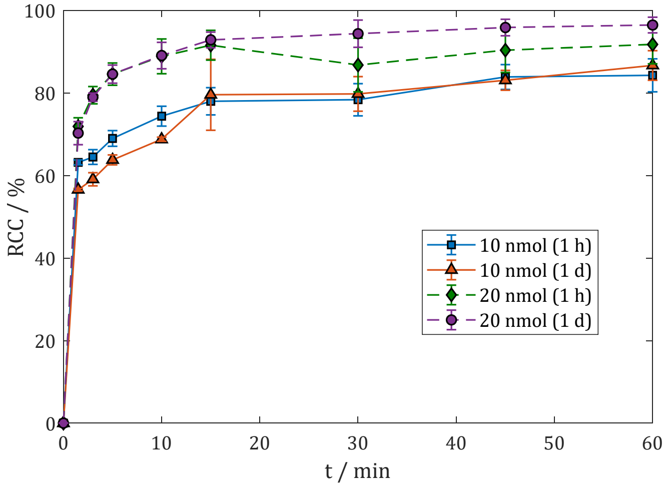

Figure S7. Reaction kinetics (radiochemical conversion (RCC) in %) of [^{225}Ac]Ac-DOTAGA.Glu.(FAPi)$_2$ **^{225}Ac-7** in 0.1 M sodium ascorbate (pH = 7.0) at 95 °C with 500 kBq ^{225}Ac (n = 2).

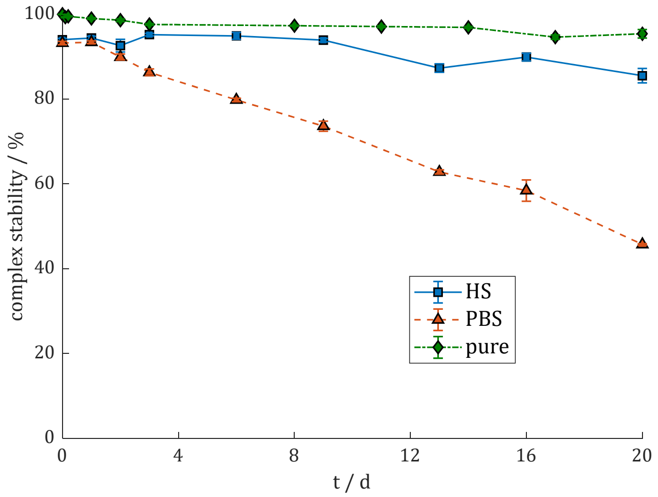

Figure S8. Complex stability of [^{225}Ac]Ac-DOTAGA.Glu.(FAPi)$_2$ **^{225}Ac-7** in human serum (HS) and phosphate-buffered saline (PBS) (0.7-0.8 MBq/mL) and final formulation(“pure”, 0.2 MBq/mL) after C18 cartridge purification over 20 days (n = 3).

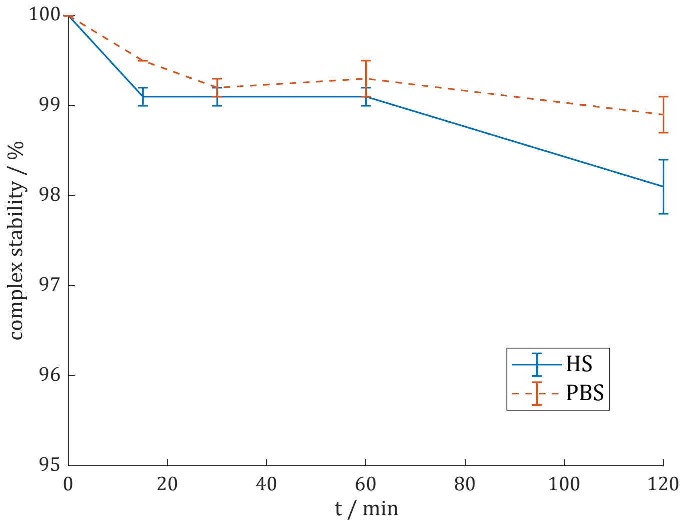

Figure S9. Complex stability of [^{68}Ga]Ga-DO3A.Glu.(FAPi)$_2$ **^{68}Ga-11** in human serum (HS) and phosphate-buffered saline (PBS) over 120 minutes (n = 3).

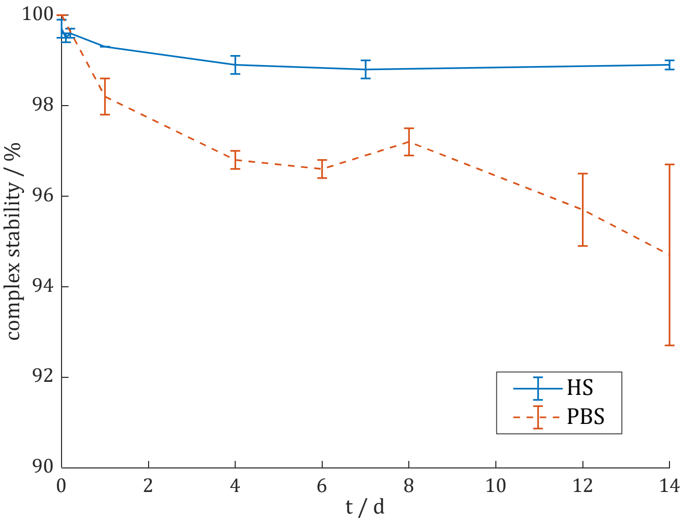

Figure S10. Complex stability of [^{177}Lu]Lu-DO3A.Glu.(FAPi)$_2$ **^{177}Lu-11** in human serum (HS) and phosphate-buffered saline (PBS) over 14 days (n = 3).

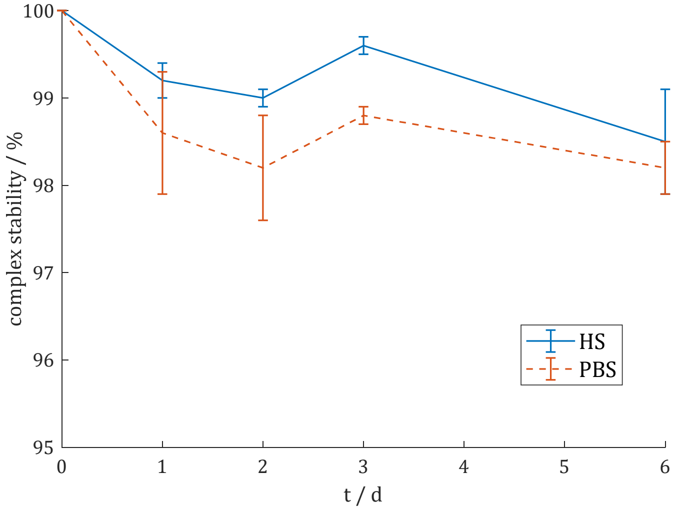

Figure S11. Complex stability of [^{90}Y]Y-DO3A.Glu.(FAPi)$_2$ **^{90}Y-11** in human serum (HS) and phosphate-buffered saline (PBS) over 6 days (n=3).

Radio-HPLC

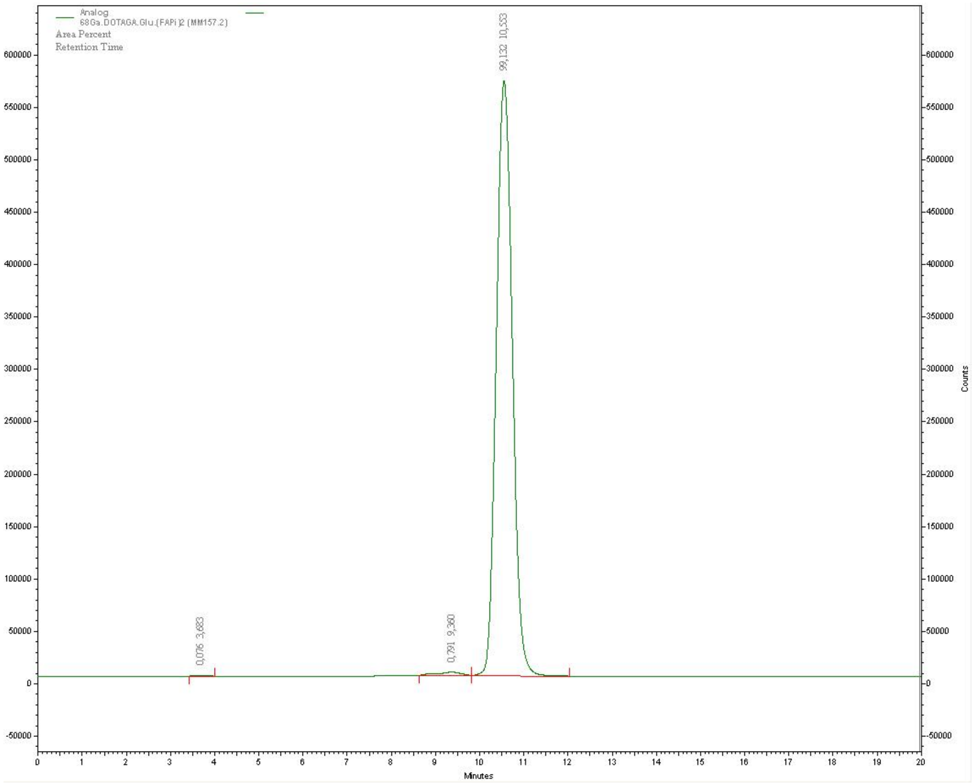

Figure S12. Analytical Radio-HPLC of [^{68}Ga]Ga-DOTAGA.Glu.(FAPi)$_2$ **^{68}Ga-7** (t_R = 10.5 min, 40 nmol **7**, 400 MBq ^{68}Ga), 20-70% MeCN + 0.1% TFA (linear gradient) in 20 min: RCP = 99.1%.

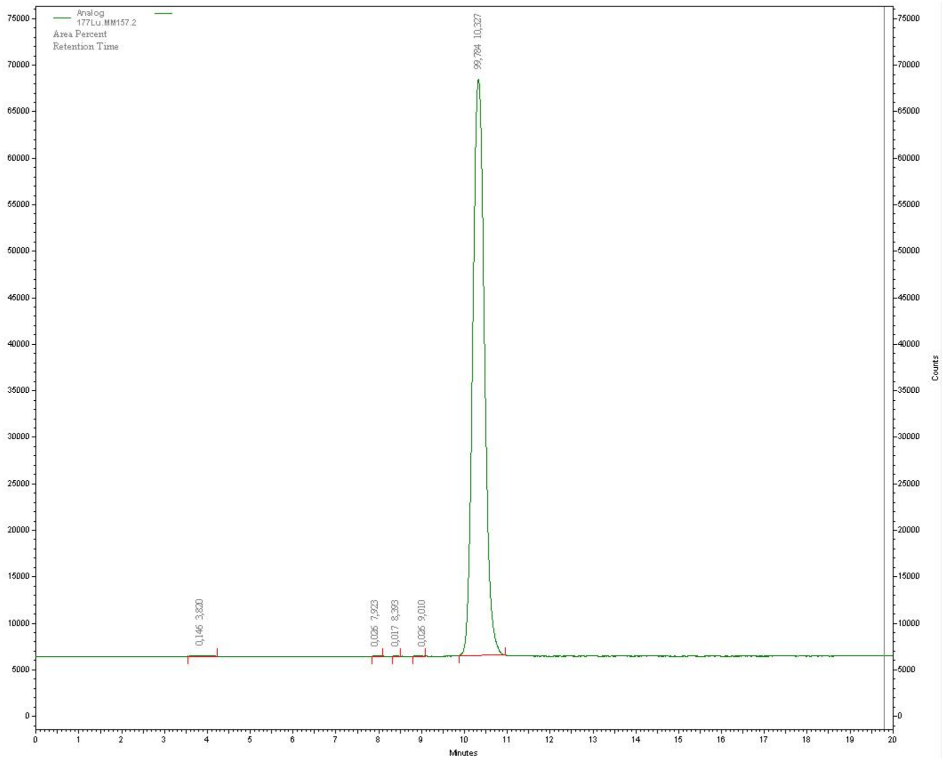

Figure S13. Analytical Radio-HPLC of [^{177}Lu]Lu-DOTAGA.Glu.(FAPi)$_2$ **^{177}Lu-7** (t_R = 10.3 min, 1 nmol **7**, 100 MBq ^{177}Lu), 20-50% MeCN + 0.1% TFA (linear gradient) in 20 min: RCP = 99.8%.

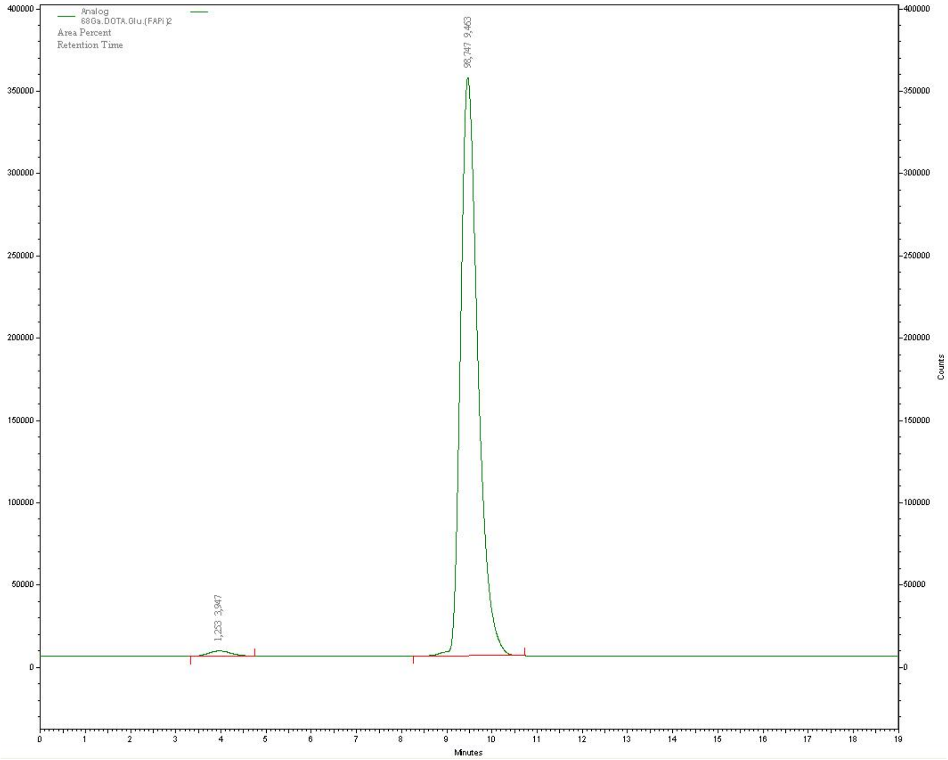

Figure S14. Analytical Radio-HPLC of [^{68}Ga]Ga-DO3A.Glu.(FAPi)$_2$ **^{68}Ga-11** (t_R = 9.5 min, 20 nmol **11**, 100 MBq ^{68}Ga), 10-90% MeCN + 0.1% TFA (linear gradient) in 20 min: RCP = 98.7%.

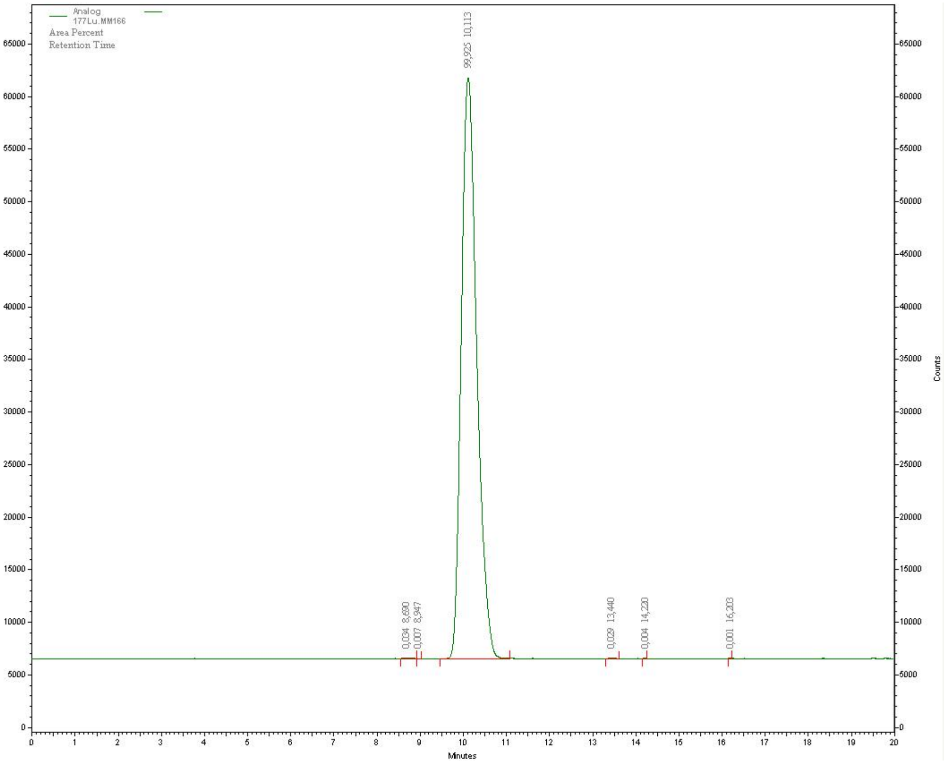

Figure S15. Analytical Radio-HPLC of [^{177}Lu]Lu-DO3A.Glu.(FAPi)$_2$ **^{177}Lu-11** (t_R = 10.1 min, 2 nmol, 100 MBq ^{177}Lu), 20-50% MeCN + 0.1% TFA (linear gradient) in 10 min: RCP = 99.9%.
